# Timing and combinations of cardiovascular diseases in survivors of childhood, adolescent, and young adulthood cancer

**DOI:** 10.1186/s40959-025-00385-8

**Published:** 2025-10-17

**Authors:** John Södling, Jakob Hytting, Panagiotis Mallios, Entela Bollano, Madeleine Johansson, Kenny A. Rodriguez-Wallberg, Elham Hedayati, Patric Karlström, Per Sundbom, Narsis Kiani, Robin Keskisärkkä, Martin Singull, Laila Hubbert

**Affiliations:** 1 Department of Cardiology Norrkoping and Department of Health, Medicine and Caring Sciences, Linkoping University, Linkoping , Sweden; 2 Department of Cardiology, Institute of Medicine at Sahlgrenska, Sahlgrenska University Hospital Gothenburg Sweden., Academy University of Gothenburg, Gothenburg, Sweden; 3 Department of Cardiology, Department of Clinical Sciences, Skane University Hospitaland, Lund University, Malmo, Sweden; 4 Department of Oncology-Pathology Karolinska Institutet, Stockholm, Sweden and Department of Reproductive Medicine, Division of Gynecology and Reproduction, Karolinska University Hospital, Stockholm, Sweden; 5 Department of Oncology-PathologyMedical Unit: BreastTheme Cancer, Karolinska InstituteEndocrine Tumors, and SarcomaKarolinska University Hospital and Comprehensive Cancer Centre, Stockholm, Sweden; 6 Department of Internal Medicine, Department of Health, Medicine and Caring Sciences, Ryhov County Hospital, Region Jonkoping County, Linkoping University, Linkoping, Sweden; 7 Department of Medicine and Geriatrics, Department of Health, Medicine and Caring Sciences, Hoglandet Hospital, Linkoping University, Linkoping, Sweden; 8 Algorithmic Dynamics Lab, Centre of Molecular Medicine, Karolinska Institute, Stockholm, Sweden; 9 Department of Computer and Information Science, Linkoping University, Linkoping, Sweden; 10 Department of Mathematics, Linkoping University, Linkoping, Sweden; 11 Department of Cardiology, Vrinnevi Hospital. Linkoping University, Norrkoping, 601 82 Sweden

## Abstract

**Background:**

Children, adolescents, and young adults with cancer (referred to as CAYAs) are at risk of long-term health complications, with cardiovascular disease (CVD) being a major concern. In addition, sociodemographic characteristics and traditional cardiovascular risk factors may also contribute to disparities in outcomes compared with those of the general population.

The aim of this study was to investigate the timing, patterns, and combinations of CVDs, as well as associated morbidity, mortality, and sociodemographic factors, in CAYAs with CVD compared with matched controls with CVD.

**Methods:**

A register-based cohort study consisting of all Swedish cancer patients under 25 years old and during a 63-year observation time was used. CAYAs and controls with CVD (*n* = 58,981) were included and compared in terms of the timing and combinations of CVD, and mortality.

**Results:**

The median age at first CVD was 41.8 years in CAYAs and 49.6 years in controls (*p* < 0.0001), with male CAYAs being the youngest at 25.0 years.

During a median follow-up of 34.6 years, most CAYAs (65.2%) developed one CVD, while two or three coexisting CVDs occurred in 20.2% and 8.2%, respectively. Mostly hypertension in combination with cerebrovascular disease, ischemic heart disease and arrhythmias.

More than three CVDs were more common in CAYAs than in controls (6.4% vs. 5.9%). A total of 21.8% of the CAYAs died, and the risk of all-cause mortality after the first CVD was 2.43-fold greater (hazard ratio (HR) 95% confidence interval (CI) 2.31–2.54, *p* < 0.0001), and for cardiovascular mortality, the risk was 2.17-fold greater (HR 95% CI 2.02–2.33, *p* < 0.0001) than that of the controls. In CAYAs with CVD, older age, male sex, and living in the central part of Sweden were associated with higher mortality, whereas higher education and marriage were protective (*p* < 0.0001).

**Conclusions:**

Compared with controls CAYAs develop advanced CVD and combinations of multiple CVDs earlier in life, and they have a greater risk of all-cause and cardiovascular mortality. Factors associated with increased mortality risk include male sex and geographic variation, whereas marriage and higher education appear to be protective.

**Graphical Abstract:**

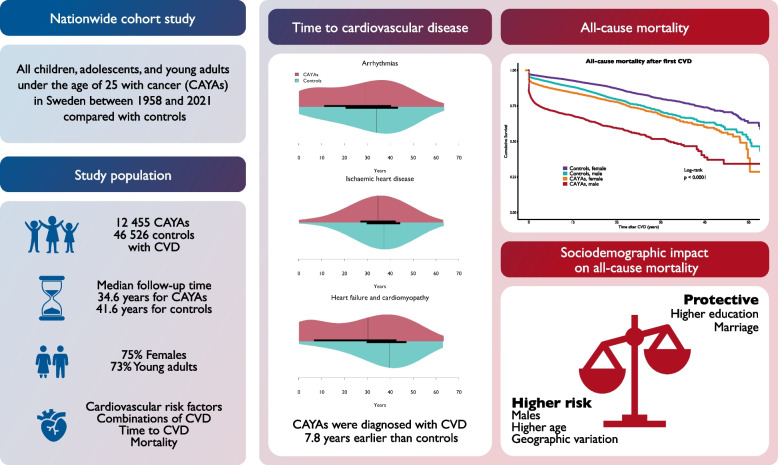

**Supplementary Information:**

The online version contains supplementary material available at 10.1186/s40959-025-00385-8.

## Introduction

The treatment of cancer in children, adolescents, and young adults (CAYA) has advanced in recent decades, improving five-year survival rates to over 80% in many European and North American countries [1]. However, survivors may face various long-term complications, including severe or potentially life-threatening diseases. These conditions may arise during cancer treatment or develop years after its completion, sometimes resulting in a lifelong and severe disease [2, 3]. Cancer survivors are affected not only by the direct impact of cancer and its treatments but also by shared risk factors and sociodemographic characteristics, which increase the risk of cardiovascular diseases (CVD) in some cases disproportionately compared with controls [4]. In the St Jude Lifetime Cohort Study, there was a 17.7% cumulative incidence of major CVD among survivors compared with 0.9% in controls [5]. The Adult Life after Childhood Cancer in Scandinavia study, reported that 8.8% of the participants had CVD requiring hospitalization. Additionally, 10% had died before reaching the age of 30 [2, 6]. Other studies have shown similar results; by the age of 60, 5.4% had developed advanced ischemic heart disease (IHD) and 11.8% had died [7–9]. The Childhood Cancer Survivor Study from the USA and Canada reported that one in five adolescents and young adults (AYA) developed CVD before they reached the age of 30 [3]. Young cancer patients have a high mortality rate following major cardiovascular events [10].

Previous reports have not covered a nation that provides universal healthcare access for all cancer and CVD patients. Data on the timing and combinations of CVDs beyond cancer treatment are also limited. Today, it remains unclear which cardiovascular and sociodemographic risk factors, in addition to the well-studied effects of cancer treatment, have the greatest impact on the timing and combination of CVD or mortality later in life.

### Objectives

The main objective of this study was to examine the timing and combinations of new-onset CVD and the risk of all-cause and cardiovascular mortality in all young Swedish cancer patients during the past six decades compared with matched controls. The aim was also to investigate socioeconomic and demographic factors associated with all-cause mortality in CAYAs who subsequently developed CVD.

## Methods

### Study design and participants

This was a register-based, matched cohort study of the total Swedish population of cancer patients under 25 years of age from January 1958 to the time of mortality or the end of the study in December 2021. In this context, the date on which cancer is diagnosed is referred to as the index. The primary study spanned a 63-year observation period, with children, adolescents, and young adults with cancer (here referred to as CAYAs) and controls matched 1:5 by age, sex, and residence at index. The study protocol for the Rebuc study (*n* = 378,108) is described elsewhere [11].

All individuals diagnosed with CVD according to the International Classification of Diseases 10th revision (ICD-10) codes I00-I99 and G45 were extracted from the primary study and included in the current study. (Supplementary data Fig. 1).

### Data retrieval

Data for all 58,981 individuals diagnosed with CVD were connected across several populations and healthcare registers. The Swedish registry system is a nationwide, high-coverage infrastructure that integrates individual-level data across multiple populations and healthcare registers using a unique personal identity number assigned to all residents. It includes comprehensive longitudinal data on diagnoses, treatments, sociodemographic factors, mortality, and health outcomes, enabling robust population-based research across decades. All registries and the variables used in this study are described in Supplemental Table 1, including the refences describing the main registries.

Driving distances to the nearest hospital at index were calculated via Google Maps API (Mountain View, CA, USA). Sweden is divided into three primary regions: northern (Norrland, with 11.4% of the population), central (Svealand, accounting for 40.8%), and southern Sweden (Gotaland, accounting for 47.8%). The southern regions have a higher population density than the northern regions do. In 2021, there were 290 municipalities, each exhibiting its own unique median income variations. The median income of the local municipality (2021) served as a proxy for both income levels and the socioeconomic status of the residential area over time. The Gini coefficient quantifies income inequality within a municipality on a scale from 0 to 1, where higher values indicate greater inequality.

Standard treatment protocols dating back to 1958 (gathered from pioneers in Swedish pediatric oncology) were employed as substitutes for treatment with radiotherapy, anthracyclines, and other medications in the treatment of leukemia, central nervous system (CNS) malignancies, Hodgkin's lymphoma, non-Hodgkin's lymphoma, and testicular cancer. No individual dose was obtained from the utilized registries.

Diagnoses were collected using ICD codes 7, 8, 9, and 10. All the data were converted to the ICD-10 (Supplementary data Table 2).

### Statistical analysis

Baseline characteristics were described via descriptive statistics, comparing CAYAs with CVD to controls with CVD. Analyses were conducted for the entire cohort and stratified by sex. Given that all the data were skewed, they are presented as the median and interquartile range (IQR). Binary and categorical variables are presented as counts (n) and percentages (%).

The subsections below outline the analyses in addition to the descriptive statistics.

The primary analysis compared CV morbidity and mortality between CAYAs and controls, with both groups being conditioned on CVD. Given this focus, a time-to-event analysis was conducted to assess when and which CVD occurred in these populations. By including only individuals who had already developed CVD, individuals who may have developed CVD later were excluded. For categorical data, between-group differences were tested for statistical significance via two-sample tests of proportions with a normal approximation. The Wilcoxon rank-sum test was applied to continuous variables when group mean differences were examined. In rare cases where the approximation was invalid, tests were omitted, and no p values were reported. For the primary and secondary outcomes, CAYAs and controls who survived five years from the index date were categorized as 5-year survivors. Subgroup analyses were conducted for the following index periods: 1958–1970, 1971–1980, 1981–1990, 1991–2000, 2001–2010, and 2011–2021.

The timing of CVD onset is presented as the time from the index to the age at CVD occurrence and was visualized in violin plots based on kernel density estimation, with the IQRs and medians indicated. Times to all-cause and CV mortality were reported as the median (IQR) years from the first CVD, whereas cumulative mortality was illustrated via Kaplan‒Meier curves by sex, age group, and index time. Survival analysis employs the date of the first CVD diagnosis as the starting point. End times were defined by all-cause or CV mortality, where applicable, or by the study endpoint in December 2021. This approach applies to secondary outcomes as well.

The frequencies of CVD combinations were reported in groups of 1, 2, or 3 conditions. The time to diagnosis for the most frequent CVD combinations is presented as the median (IQR) years from the index.

Survival analyses were performed via Cox regression models to assess hazard ratio (HR) with 95% confidence interval (CI) for CAYAs and controls across various factors and covariates. Univariable and multivariable Cox models were used to evaluate the influence of sociodemographic factors on all-cause mortality. In the multivariable model, all variables were entered simultaneously, adjusting for matched control variables. To control for early morbidity and mortality, an analysis was conducted on participants aged 30 years or older at the study endpoint, with a focus on educational level, and civil status., and sick leave. Martingale and Schoenfeld residuals were used to assess the assumptions of the Cox regression models, and no major violations were found. Interactions between subgroups with different covariates were tested.

The *p* value is used as a continuous measure to indicate the strength of evidence along with the HR and 95% CI. Analyses were performed via R version 4.3.3 (R Foundation for Statistical Computing, Vienna, Austria).

## Results

The baseline and sociodemographic data are presented in Table 1. A total of 58,981 individuals with CVD were included: 12,455 CAYAs (71.8% females) and 46,526 controls (76.2% females), accounting for 387,000 patient-years in CAYAs and 1.8 million patient-years in controls. The median follow-up period for CAYAs was 34.6 years (IQR 15.4–45.5), and that for controls was 41.6 years (30.9–49.0) (*p* < 0.0001). At study completion, the median age of CAYAs reached 53.9 years (IQR 32.5–66.1), whereas the median age of controls was 60.9 years (49.6–69.2) (*p* < 0.0001). Significant differences in sex distribution were observed, influenced by index cancer types, with most females being in the older age group among both CAYAs and controls (76.4% vs. 83.6%). The most common index cancer was cervical cancer, followed by CNS tumors and leukemia.

A majority of CAYAs received their index cancer diagnosis prior to 1991 and were younger than controls were at the index (median age 21 years, IQR 16–23 vs. 22 years, IQR 19–23 *p* < 0.0001).

### Sociodemographic factors in CAYAs

**Fig. 1:**
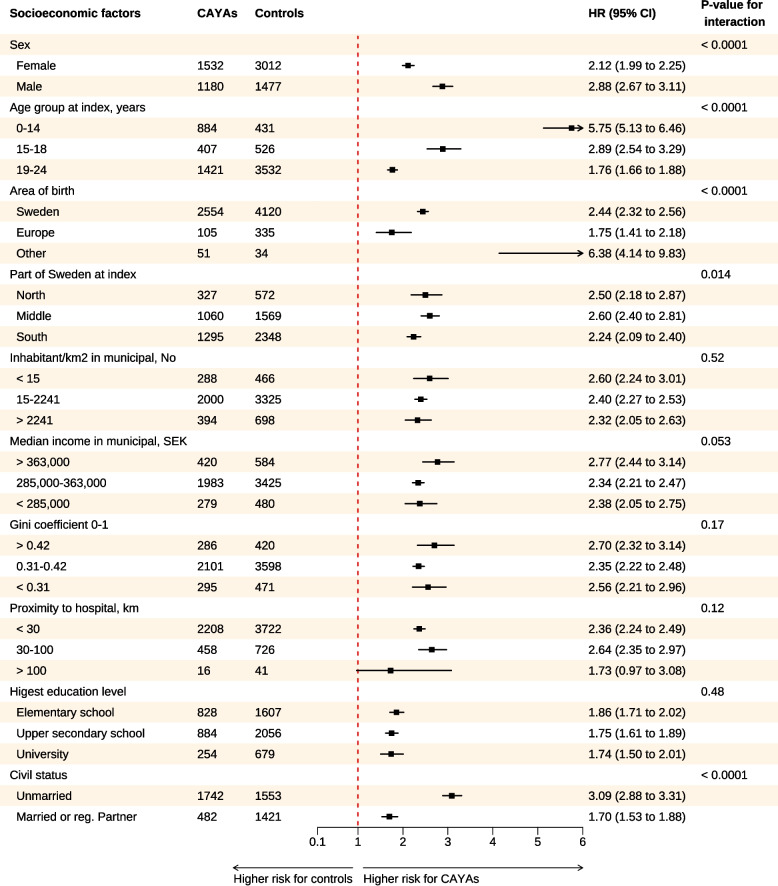
Forest plot visualizing subgroup analysis via Cox regression. Hazard ratios for all-cause mortality were calculated between children, adolescents, and young adults with index cancer and controls across subgroups after the first CVD. Abbreviations: CAYA = children, adolescents, and young adults, CVD = cardiovascular disease, CI = confidence interval, HR = hazard ratio

At index, most CAYAs and controls were born in Sweden (93.9% vs. 92.3%, *p* < 0.0001), and the majority lived in southern parts (*p* < 0.0001). At index 9.4% CAYAs and 9.7% controls lived in a rural municipality and 13.4% vs. 13.1% in a high-density urban area. (Table 1 and Fig. 1) CAYAs were more likely to reside in municipalities with a high median income, 14.2%, than in those with a low median income, 12.9% (*p* = 0.0009). However, the GINI coefficient revealed no disparity between the groups. Similarly, the distance to the nearest hospital was comparable for both CAYAs and controls, and the majority lived < 30 km from a hospital (Supplementary data Table 7).

**Table 1:** Sociodemographic factors and clinical characteristics

	**CAYAs**	**Controls**	*p***-value**^**a**^
n (%)	12 455	46 526	
Age at index years median (IQR)	21 (16-23)	22 (19-23)	
Females n (%)	8947 (71.8)	35 449 (76.2)	
**Age group at index** n (%)			< 0.0001
0–14	2717 (21.8)	6497 (14.0)	
15–18	1534 (12.3)	5074 (10.9)	
19–24	8204 (65.9)	34 955 (75.1)	
**Decade at index** n (%)			< 0.0001
1958–1970	2040 (16.4)	9892 (21.3)	
1971–1980	3364 (27.0)	15 659 (33.7)	
1981–1990	2518 (20.2)	10 198 (21.9)	
1991–2000	1660 (13.3)	5362 (11.5)	
2001–2010	1427 (11.5)	3245 (7.0)	
2011–2021	1446 (11.6)	2170 (4.7)	
**Index cancer diagnosis** n (%)			
Female reproduction organ^b^	6207 (49.8)		
CNS tumors	1242 (10.0)		
Leukemia	1189 (9.6)		
Lymphoma	839 (6.7)		
Thyroid and endocrine glands	562 (4.5)		
Male reproductive organs	393 (3.2)		
Skin	382 (3.1)		
Gastrointestinal	317 (2.6)		
Bone tumors	298 (2.4)		
Kidney and urorenal tract	249 (2.0)		
Soft tissue sarcoma	224 (1.8)		
Eye	71 (0.6)		
Breast	39 (0.3)		
Lung	39 (0.3)		
Other malignancies	404 (3.2)		
**Cancer treatment**^**c**^ n (%)	3330 (26.7)		
Radiotherapy	1976 (59.3)		
Anthracycline	1656 (49.7)		
Other drugs	2706 (21.7)		
**Area of birth**^**d**^ n (%)			< 0.0001
Sweden	11 693 (93.9)	42 918 (92.3)	
Europe	534 (4.3)	2727 (5.9)	
Other^d^	226 (1.8)	881(1.9)	
**Part of Sweden at index** n (%)			< 0.0001
North	1388 (11.2)	5487 (11.8)	
Central	4291 (34.5)	15 172 (32.6)	
South	6744 (55.6)	25 867 (55.6)	
**Highest level of Education** n (%)			< 0.0001
Elementary school 9 years	2353 (18.9)	7523 (16.2)	
Upper secondary school	5517 (44.3)	22 120 (47.5)	
University	3253 (26.1)	15 708 (33.8)	
University > 30 years of age	2980 (31.0)	15 357 (35.0)	
Postgraduate education	71 (0.6)	376 (0.8)	
**Civil status** n (%)			< 0.0001
Unmarried	5472 (43.9)	13 178 (28.3)	
Married or registered partner	4222 (33.9)	21 573 (46.4)	
Married > 30 years of age	4132 (33.2)	21 373 (45.9)	
Na	2761 (22.2)	11 775 (25.3)	
Median age at study end (IQR)	53.9 (32.5–66.1)	60.9 (49.6–69.2)	< 0.0001

12,455 child, adolescent, and young adult patients with cancer and cardiovascular disease and 46,526 controls encompassing all cancer patients in Sweden under the age of 25 years from January 1958 to December 2021.Patients included were between < 1 years and 24 years at the time of their index cancer diagnosis with 1:5 matched controls based on age sex and place of residence

*Abbreviations: CAYAs* children adolescents and young adults, *CNS *central nervous system, *IQR *interquartile range. ·· not applicable. *n* numbers, *NA* not available

^a^ *p*-values apply to comparisons between all CAYAs and controls for outcomes

^b^ Including cervical intraepithelial neoplasia (CIN) and high-grade squamous intraepithelial lesion (HSIL)

^c^ Treatment for leukemia, CNS malignancies, lymphoma, and testis cancer according to standard protocols

^d^ Other = North America 0.08% South America 0.3% Africa 0.3% Asia 1.2% Russia 0.02% Oceanian 0.01% and unknown < 0.01%

### Cardiovascular risk factors in CAYAs

All the cardiovascular risk factors (CVRF) are listed in Table 2. CVRFs were found in 5501 (44.2%) CAYAs with incident CVD compared with 22,239 (47.8%) controls (*p* < 0.0001). The most common CVRF in CAYAs was dyslipidemia (31.0%), followed by diabetes mellitus (13.1%), hypothyroidism (8.7%), chronic obstructive pulmonary disease (6.5%), and chronic kidney disease (3.6%) (all *p* < 0.0001).

More CAYAs had a history of smoking (65.4% vs 58.2% in controls, *p* < 0.0001), particularly female ever smokers (71.2%). In comparison, 13.6% reported having ever used snuff, with a greater proportion of female snuff users among CAYAs than among the control group (7.7% vs. 6.8%, *p* = 0.020). Obesity was observed in 36.3% of CAYAs and 41.2% of controls (*p* < 0.0001).

### Timing of first-onset CVD in CAYAs

**Fig. 2:**
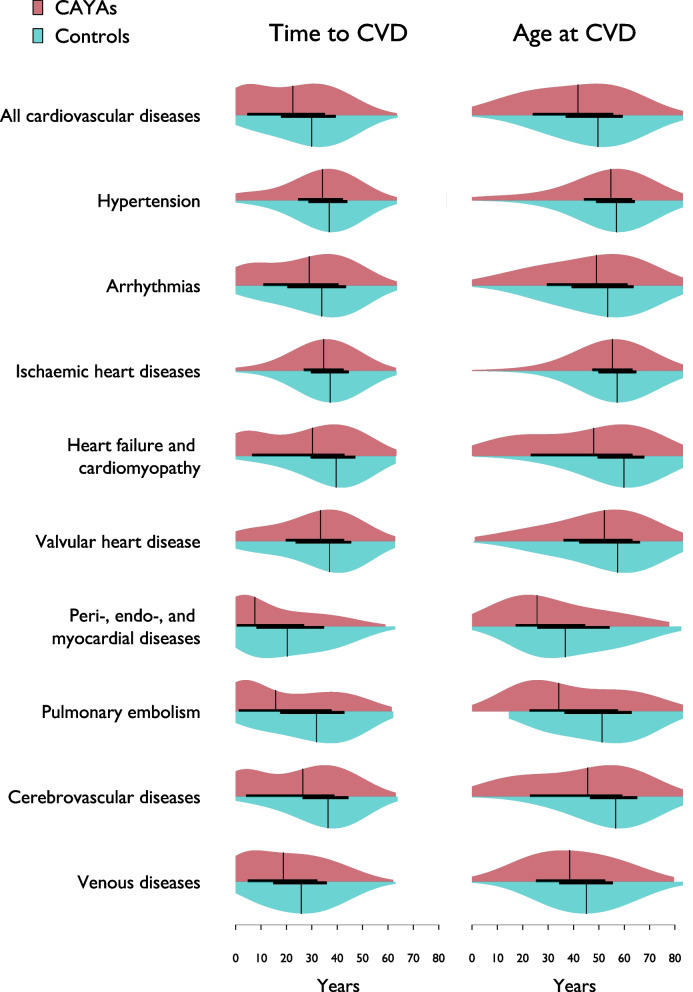
Violin plots comparing time to CVD occurrence and age at CVD diagnosis between CAYAs and controls. The plots illustrate the distribution, density, and variability in the timing and age of CVD onset across the two groups. Abbreviations: CAYA = children, adolescents, and young adults. CVD = cardiovascular disease

The frequencies, timings and combinations of CVDs are listed in Table 2 and illustrated in Fig. 2.

**Table 2:** Age at onset and time to cardiovascular diseases

	**CAYAs**			**Controls**			
	Female	Male	**Total**	Female	Male	**Total**	*p*-value^a^
n (%)	8947 (71.8)	3508 (28.2)	12 455	35 449 (76.2)	11 077 (23.8)	46 526	
**Cardiovascular risk factors** n (%)	4300 (48.1)	1201 (34.2)	5501 (44.2)	16 838 (47.5)	5401 (48.8)	22 239 (47.8)	< 0.0001
Diabetes mellitus	1244 (13.9)	383 (10.9)	1627 (13.1)	5198 (14.7)	2072 (18.7)	7270 (15.6)	< 0.0001
Chronic Kidney Disease	276 (3.1)	168 (4.8)	444 (3.6)	862 (2.4)	387 (3.5)	1249 (2.7)	< 0.0001
Hyperlipidaemia	3016 (33.7)	840 (23.9)	3856 (31.0)	12 842 (36.2)	4445 (40.1)	17 287 (37.2)	< 0.0001
Hypothyroidism	871 (9.7)	215 (6.1)	1086 (8.7)	2868 (8.1)	170 (1.5)	3038 (6.5)	< 0.0001
Hyperthyroidism	276 (3.1)	25 (0.7)	301 (2.4)	1151 (3.2)	83 (0.8)	1234 (2.7)	0.052
Chronic obstructive pulmonary disease	740 (8.3)	75 (2.1)	815 (6.5)	1821 (5.1)	325 (2.9)	2146 (4.6)	< 0.0001
Interstitial pulmonary disease	75 (0.8)	48 (1.4)	123 (1.0)	190 (0.5)	73 (0.7)	263 (0.6)	< 0.0001
Ever smoker^b^	1349 (71.2)	292 (47.5)	1641(65.4)	4606 (58.1)	2044 (58.4)	6650 (58.2)	< 0.0001
Ever snuffer^b^	91 (7.7)	124 (31.6)	215 (13.6)	346 (6.8)	823 (36.8)	1169 (15.9)	0.020
Obesity^c^ (BMI > 30 kg/m2)	587 (37.4)	158 (32.6)	745 (36.3)	2817 (42.4)	1105 (38.4)	3922 (41.2)	< 0.0001
**Cardiovascular diseases** n (%) **Time to CVD and Age** years (IQR)							< 0.0001
**Any CVD**	8947	3508	12 455	35 449	11 077	46 526	< 0.0001
Time from index to CVD	26.1 (9.6–36.7)	9.0 (0.6–27.9)	22.5 (4.7–35.0)	29.7 (17.8–38.9)	30.8 (18.9–40.7)	29.9 (18.0–39.3)	< 0.0001
Median age at CVD	46.8 (29.7–57.8)	25.0 (16.1–43.9)	41.8 (24.1–55.5)	50.4 (38.0–59.8)	47.1 (34.1–57.2)	49.6 (37.1–59.2)	< 0.0001
**Hypertension (HTN)**	3818 (42.7)	1103 (31.4)	4921 (39.5)	16,436 (46.4)	5026 (45.4)	21,462 (46.1)	< 0.0001
Time to HTN	35.2 (27.3–42.5)	27.9 (9.5–39.9)	34.2 (24.8–42.1)	36.6 (28.7–43.5)	37.9 (29.3–45.0)	36.8 (28.8–43.8)	< 0.0001
**Arrhythmias (ARY)**	1898 (21.2)	706 (20.1)	2604 (20.9)	7866 (22.2)	3138 (28.3)	11 004 (23.7)	< 0.0001
Time to ARY	30.6 (13.8–41.1)	22.4 (3.9–38.2)	29.0 (11.1–40.4)	33.6 (19.8–42.9)	34.9 (22.4–44.4)	33.9 (20.5–43.3)	< 0.0001
**Atrial fibrillation and flutter (AF)**	674 (7.5)	281 (8.0)	955 (7.7)	2889 (8.2)	1644 (14.8)	4533 (9.7)	< 0.0001
Time to AF	40.1 (32.7–47.1)	35.9 (24.9–44.8)	39.2 (30.4–46.2)	41.5 (33.5–47.7)	38.9 (29.4–46.6)	40.7 (32.0–47.5)	0.001
**Ischemic heart diseases (IHD)**	1080 (12.1)	406 (11.6)	1486 (11.9)	4058 (11.5)	2200 (19.9)	6258 (13.5)	< 0.0001
Time to IHD	35.5 (28.5–42.9)	32.1 (22.2–40.8)	34.7 (27.0–42.3)	36.8 (29.4–43.9)	38.0 (30.5–45.2)	37.2 (29.8–44.4)	< 0.0001
**Acute myocardial infarction (MI)**	571 (6.4)	230 (6.6)	801 (6.4)	1976 (5.6)	1289 (11.6)	3265 (7.0)	0.022
Time to MI	36.7 (29.5–43.5)	33.4 (21.6–42.3)	36.1 (27.7–43.3)	37.5 (29.9–45.5)	38.6 (30.8–45.4)	37.9 (30.3–45.5)	< 0.0001
**Heart failure and cardiomyopathy (HF)**	806 (9.0)	468 (13.0)	1274 (10.2)	2251 (6.3)	1137 (10.0)	3388 (7.3)	< 0.0001
Time to HF	34.0 (15.4–44.3)	18.0 (1.9–36.0)	30.2 (6.7–42.7)	40.0 (30.1–47.2)	38.9 (29.1–46.9)	39.6 (29.7–47.0)	< 0.0001
**Valvular heart disease (VHD)**	368 (4.1)	248 (7.1)	616 (5.0)	1462 (4.1)	623 (5.6)	2085 (4.5)	0.028
Time to VHD	35.1 (21.5–44.4)	32.1 (19.1–38.9)	33.4 (19.9–42.5)	37.2 (23.7–45.1)	36.6 (24.2–45.5)	37.0 (23.8–45.3)	< 0.0001
**Peri-, endo-, and myoc. diseases**	285 (3.2)	282 (7.8)	567 (4.6)	860 (2.4)	714 (6.5)	1574 (3.4)	< 0.0001
Time to Peri endo and myoc. diseases	14.5 (1.3–31.7)	4.3 (0.3–19.1)	7.5 (0.7–26.7)	24.8 (9.5–38.0)	17.0 (7.3–29.7)	20.3 (8.3–34.6)	< 0.0001
**Pulmonary embolism (PE)**	455 (5.1)	247 (7.0)	702 (5.6)	1462 (4.1)	445 (4.0)	1907 (4.1)	< 0.0001
Time to PE	23.8 (4.6–41.0)	2.6 (0.3–28.2)	15.7 (1.4–37.6)	30.5 (15.8–42.4)	35.3 (23.9–44.2)	31.8 (17.7–42.7)	< 0.0001
**Cerebrovascular diseases**	1585 (17.7)	762 (21.7)	2347 (18.8)	4660 (13.2)	1569 (14.1)	6229 (13.4)	< 0.0001
Time to Cerebrovascular diseases	30.2 (11.6–40.1)	12.1 (0.7–33.7)	26.4 (4.2–38.7)	36.0 (26.2–44.0)	37.5 (27.6–45.4)	36.4 (26.5–44.3)	< 0.0001
**Diseases of veins (VD)**	2105 (23.5)	721 (20.6)	2826 (22.7)	9012 (25.4)	1969 (17.8)	10,981 (23.6)	0.033
Time to VD	22.1 (9.5–34.0)	5.8 (0.4–23.5)	18.8 (4.9–32.1)	25.5 (14.6–35.3)	27.6 (16.2–38.2)	25.8 (14.9–35.8)	< 0.0001
**Other CVD**	807 (9.0)	383 (10.9)	1190 (9.5)	2642 (7.5)	1033 (9.3)	3675 (7.9)	< 0.0001
Time to Others	18.8 (3.5, 36.2)	9.3 (0.9, 29.3)	15.4 (2.5, 34.5)	23.7 (7.4, 39.2)	27.4 (8.7, 42.6)	24.6 (7.8, 40.0)	< 0.0001
**1. Only one CVD** n (%)	5820 (65.0)	2299 (65.5)	8119 (65.2)	23 642 (66.7)	6672 (60.9)	30 314 (65.2)	
1:1 (%)	HTN (29.8)	HTN (20.5)	HTN (27.2)	HTN (33.5)	HTN (30.0)	HTN (32.6)	
Time to 1:1 years median (IQR)	33.2 (24.3–40.8)	19.5 (0.9–33.6)	31.6 (19.8–40.1)	34.7 (26.8–41.8)	35.3 (25.5–43.1)	34.8 (26.5–42.1)	
1:2 (%)	VD (21.3)	CVA (17.7)	VD (20.2)	VD (24.9)	VD (18.0)	VD (23.2)	
Time to 1:2 years median (IQR)	18.0 (7.0–28.9)	2.2 (0.2–19.8)	15.0 (3.7–27.2)	22.7 (12.7)	23.6 (13.3–34.2)	23.0 (12.8–32.7)	
1:3 (%)	ARY (12.4)	VD (17.5)	CVA (13.0)	ARY (14.4)	ARY (17.5)	ARY (15.4)	
Time to 1:3 years median (IQR)	15.8 (4.3–29.8)	3.6 (0.4–17.4)	8.6 (0.8–29.4)	24.3 (10.7–36.2)	23.0 (12.1–34.8)	23.8 (11.0–36.0)	
**2. Combination of two CVD** n (%)	1824 (20.4)	693 (19.8)	2517 (20.2)	7296 (20.6)	2364 (21.3)	9660 (20.8)	
2:1 (%)	HTN/CVA (14.3)	HTN/CVA (11.4)	HTN/CVA (13.5)	HTN/ARY (14.6)	HTN/IHD (17.6)	HTN/ARY (14.5)	
Time to 2:1 years median (IQR)	39.5 (31.1–44.7)	37.9 (18.3–46.4)	39.1 (29.9–45.0)	40.0 (33.2–46.0)	41.1 (33.3–47.3)	40.3 (33.7–46.8)	
2:2 (%)	HTN/IHD (11.5)	HTN/IHD (8.9)	HTN/IHD (10.8)	HTN/CVA (14.3)	HTN/ARY (14.3)	HTN/CVA (13.9)	
Time to 2:2 years median (IQR)	38.8 (32.9–44.3)	33.8 (25.1–43.5)	38.2 (31.4–44.3)	40.9 (33.8–46.7)	42.4 (34.2–48.0)	40.9 (33.8–46.9)	
2:3 (%)	HTN/ARY (10.9)	HTN/ARY (7.6)	HTN/ARY (10.0)	HTN/IHD (12.7)	HTN/CVA (13.0)	HTN/IHD (13.9)	
Time to 2:3 years median (IQR)	40.3 (33.8–45.9)	30.4 (18.1–44.6)	39.7 (30.4–45.4)	39.4 (32.6–45.6)	40.9 (33.8–47.1)	40.1 (32.8–46.2)	
**3. Combination of three CVD** n (%)	747 (8.3)	277 (14.1)	1024 (8.2)	2697 (7.6)	1122 (10.1)	3819 (8.2)	
3:1 (%)	HTN/ARY/CVA (7.8)	HTN/ARY/CVA (6.5)	HTN/ARY/CVA (7.4)	HTN/IHD/ARY (9.3)	HTN/IHD/ARY (11.5)	HTN/IHD/ARY (10.0)	
Time to 3:1 years median (IQR)	43.9 (38.1–52.7)	40.2 (36.1–47.5)	42.7 (37.3–50.4)	44.0 (37.3–49.1)	44.2 (36.7–50.6)	44.1 (37.2–49.7)	
3:2 (%)	HTN/ARY/HF (6.4)	HTN/IHD/ARY (4.7)	HTN/IHD/ARY (5.7)	HTN/ARY/CVA (6.1)	HTN/ARY/HF (7.4)	HTN/ARY/CVA (6.3)	
Time to 3:2 years median (IQR)	42.3 (36.0–49.2)	39.5 (33.6–45.1)	43.7 (36.6–50.2)	44.3 (38.6–50.4)	43.1 (34.2–48.6)	44.6 (38.6–50.7)	
3:3 (%)	HTN/ARY/IHD (6.0)	HTN/CVA/VD (4.3)	HTN/ARY/HF (5.6)	HTN/ARY/HF (6.0)	HTN/ARY/CVA (6.8)	HTN/ARY/HF (6.3)	
Time to 3:3 years median (IQR)	44.8 (37.2–50.4)	43.1 (32.3–50.6)	41.6 (33.5–48.8)	45.7 (37.9–50.5)	45.7 (38.6–50.8)	44.5 (37.2–50.1)	
** > 3 CVD** n (%)	556 (6.2)	239 (6.8)	795 (6.4)	1814 (5.1)	919 (8.3)	2733 (5.9)	

12,455 child, adolescent, and young adults with cancer and cardiovascular disease and 46,526 controls, encompassing all patients in the Swedish National Cancer Register under the age of 25 years from January 1958 to December 2021. *Abbreviations: ArtCap* Arteries, arterioles and capillaries diseases, *ARY* Arrhythmias, *CAYAs* children, adolescents, and young adults, *CVA* cerebrovascular accident, *HF* Heart failure, *HTN *Hypertension, *IHD* ischemic heart disease, *IQR* interquartile range, *VD* Diseases of veins

^a^ *p*-values apply to comparisons between all CAYAs and controls, regardless of sex

^b^ Non available data for smoking 9,944 CAYAs and 39,188 controls and for snuffing 10,873 vs 39,118

^c^ Non available data for BMI 10 402 CAYAs and 37,007 controls

The median time to develop CVD from the index was shorter for CAYAs than for controls (22.5 years, IQR 4.7–35.0 vs. 29.9 years, IQR 18.0–39.3, *p* < 0.0001). The CAYAs were 7.8 years younger than the controls when they presented with their first CVD (41.8 vs. 49.6 years, *p* < 0.0001), with male CAYAs being the youngest at 25.0 years.

Hypertension was the most common CVD in CAYAs (39.5%), identified after a median of 34.2 years (IQR 24.8–42.1) post-index and occurring 2.6 years earlier than in controls (*p* < 0.0001). The second most common CVD in CAYAs was diseases of veins (22.7%), mostly varicose veins (9.6%), followed by venous embolism and thrombosis (4.3%). These conditions emerged after 18.8 years (IQR 4.9–32.1) post-index in CAYAs and 7.0 years earlier than in controls (*p* < 0.0001). Arrhythmias, ranked as the third most common incident CVD in CAYAs (20.9%), occurred 4.9 years earlier than in controls (*p* < 0.0001). IHD was found in 11.9% of CAYAs, with a median age of 55.4 years (IQR 47.5–63.1), and appeared 2.5 years earlier than it did in controls (*p* < 0.0001). Heart failure occurred in 10.2% of CAYAs, with a median age of 48.0 years (IQR 23.3–63.1), and occurred 9.4 years earlier than in controls (*p* < 0.0001). Pericardial, endocardial, and myocardial diseases, as well as cerebral hemorrhage, which are known complications of both childhood cancer and treatment, affected the youngest CAYAs. Time to and age to CVD amongst the youngest females and males with Leukemia, CNS malignancies, Lymphoma, and Testis cancer that received treatment according to standard protocols are listed in the Supplemental data Table 5 a-c.

### Co-occurrence CVDs in CAYAs

The majority of CAYAs (65.2%) experienced only one CVD. Approximately one-fifth of CAYAs presented with two coexisting incident CVDs, mostly hypertension combined with cerebrovascular disease, followed by hypertension combined with ischemic heart disease and hypertension combined with arrhythmias.

The co-occurrence of three CVDs occurred in 8.2% of the CAYAs, mainly hypertension combined with arrythmias and cerebrovascular disease. More than three CVDs were more common in CAYAs than in controls (6.4% vs. 5.9%). The co-occurrence of more than one CVD consistently occurred earlier in CAYAs than in controls (Table 2).

### Mortality among CAYAs with CVD

**Fig. 3:**
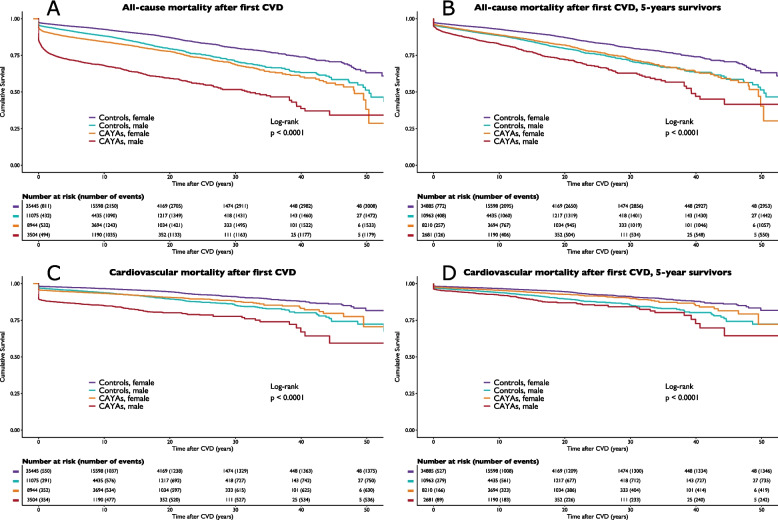
Kaplan Meier curves comparing survival outcomes after the first cardiovascular disease across four groups: female CAYAs, male CAYAs, female controls, and male controls. The log-rank test was used to test statistical significance. The panels depict (**A**) all-cause mortality; (**B**) all-cause mortality restricted for 5-year survivors following index cancer; (**C**) cardiovascular mortality; and (**D**) cardiovascular mortality restricted to 5-year survivors following index cancer. Abbreviations: CAYA = Children, adolescents, and young adults

The mortality rates are presented in Table 3 and Fig. 3. After a median follow-up of 7.4 years (IQR 2.3–14.1) after the first CVD diagnosis, 2714 (21.8%) CAYAs died. Compared with that of controls, the risk of all-cause mortality after the first CVD was 2.43-fold greater (HR 95% CI 2.31–2.54, p < 0.0001), whereas survival was 30.6 years shorter for CAYAs. The median time to mortality after the first CVD was 0.7 years (IQR 0.0–7.0) for female CAYAs and 0.2 years (0.0–2.8) for male CAYAs. Five years after the first CVD, 21.1% of the CAYAs and 7.6% of the controls had died.

**Table 3:** All cause and cardiovascular mortality outcomes after the first cardiovascular disease

	**CAYAs**			**Controls**			
	Female	Male	**Total**	Female	Male	**Total**	*p*-value^a^
n (%)	8947 (15.2)	3508 (5.9)	12 455 (21.1)	35 449 (60.1)	11 077 (18.8)	46 526 (78.9)	
Follow-up time after CVD years, median (IQR)	7.9 (2.8–14.5)	5.6 (0.9–12.8)	7.4 (2.3–14.1)	8.7 (3.8–14.9)	7.9 (3.3–14.0)	8.5 (3.7–14.7)	< 0.0001
**All-cause mortality** ^b^	HR 2.43 (HR 2.31–2.54, p < 0.0001)						
All-cause mortality n (%)	1534 (17.1)	1180 (33.6)	2714 (21.8)	3013 (8.5)	1478 (13.3)	4491 (9.7)	< 0.0001
Age median (IQR)	46.7 (20.4–63.6)	22.9 (13.9–44.4)	29.8 (16.7–58.5)	61.5 (50.6–69.3)	58.1 (46.9–66.7)	60.4 (49.4–68.5)	< 0.0001
Years after first CVD median (IQR)	0.7 (0.0–7.0)	0.2 (0.0–2.8)	0.4 (0.0—11.2)	3.4 (0.0–11.2)	2.8 (0.0–10.7)	3.2 (0.0–11.1)	< 0.0001
5-year mortality after CVD n (%) ^c^	1072 (15.8)	954 (34.2)	2026 (21.1)	1713 (6.6)	872 (10.8)	2585 (7.6)	< 0.0001
> 5 years after index n (%)	1058 (12.9)	551 (20.6)	1609 (14.8)	2958 (8.5)	1448 (13.2)	4406 (9.6)	< 0.0001
> 5 years after index median age (IQR)	58.0 (44.4–66.8)	46.2 (30.6–60.3)	54.8 (38.8–65.2)	61.9 (51.4–69.4)	58.7 (47.7–66.8)	60.7 (50.1–68.7)	< 0.0001
> 5 years after index 5-year mortality after CVD n (%) ^c^	596 (9.4)	325 (15.0)	921 (10.8)	1658 (6.4)	842 (10.4)	2500 (7.3)	< 0.0001
**Cardiovascular mortality **^b^	HR 2.17 (HR 2.02–2.33, p < 0.0001)						
Cardiovascular mortality n (%)	630 (7.0)	537 (15.3)	1167 (9.3)	1378 (3.9)	754 (6.8)	2132 (4.6)	< 0.0001
Age median (IQR)	44.6 (17.1–61.1)	21.7 (10.9–47.5)	28.7 (14.2–57.1)	60.0 (49.6–68.2)	58.1 (47.5–66.2)	59.3 (48.8–67.5)	< 0.0001
Years after first CVD median (IQR)	0.0 (0.0–4.6)	0.0 (0.0–0.9)	0.0 (0.0–2.8)	0.8 (0.0–10.0)	1.2 (0.0–9.3)	1.0 (0.0–9.7)	< 0.0001
5-year CV mortality after CVD n (%)^e^	479 (7.7)	447 (19.6)	926 (10.9)	880 (3.5)	476 (6.2)	1356 (4.1)	< 0.0001
> 5 years after index n (%)	419 (5.1)	243 (9.1)	662 (6.1)	1349 (3.9)	739 (6.7)	2088 (4.6)	< 0.0001
> 5 years after index median age (IQR)	56.8 (44.9–65.1)	50.0 (33.4–61.0)	54.8 (41.3–64.1)	60.4 (50.7–68.4)	58.6 (48.2–66.4)	59.7 (49.6–67.7)	< 0.0001
> 5 years after index 5-year mortality after CVD n (%)^f^	268 (4.5)	153(7.7)	421 (5.3)	851 (3.4)	461 (6.0)	1312 (4.0)	< 0.0001

12,455 child, adolescent, and young adult patients with cancer and cardiovascular disease, encompassing all cancer patients in Sweden under the age of 25 years from January 1958 to December 2021, compared to controls. *Abbreviations: CAYAs* children, adolescents, and young adults, *CV* cardiovascular, *CVD* cardiovascular diseases, *HR* hazard ratio, *IQR* interquartile range, *Na* not applicable, *n* numbers

^a^ *p*-values apply to comparisons between all CAYAs and controls, regardless of sex

^b^ HR calculated using cox proportional hazards regression model comparing CAYAs with controls for all-cause mortality and cardiovascular mortality

^c^ 2861 CAYAs (2142 female and 719 male) and 12,339 controls (9366 female and 2973 male) censored due to follow-up shorter than 5 years

^d^ 2403 CAYAs (1882 female and 521 male) and 11,752 controls (8861 female and 2891 male) censored due to follow-up shorter than 5 years

^e^ 3961 CAYAs (2735 female and 1226 male) and 13,568 controls (10,199 female and 3369 male) censored due to follow-up shorter than 5 years,or mortality from other cause

^f^ 2903 CAYAs (2210 female and 693 male) and 12,940 controls (9668 female and 3272 male) censored due to follow-up shorter than 5 years, or mortality from other cause

CAYAs that survived five years after the index had a life expectancy that was 5.9 years shorter than that of the controls (female CAYAs 3.9 years earlier, whereas male CAYAs 12.5 years earlier than controls, *p* < 0.0001). The median age at death for five-year survivors was 58 years (IQR 44.4–66.8) for female CAYAs and 46.2 years (30.6–60.3) for males. Compared with male controls, male CAYAs experienced the highest all-cause mortality after the first CVD (HR 2.88 95% CI 2.67–3.11, *p* < 0.0001) and cardiovascular mortality (HR 2.51 95% CI 2.24–2.80, *p* < 0.0001).

Cardiovascular mortality occurred in 9.3% of CAYAs, with death occurring 1.0 years earlier after CVD than in controls (*p* < 0.0001). Compared with that of controls, the risk of cardiovascular mortality after the first CVD was 2.17-fold (HR 95% CI 2.02–2.33, *p* < 0.0001) greater in CAYAs. Among CAYA five-year survivors, cardiovascular mortality occurred 4.9 years earlier in life (females 3.6 years and 8.6 years for males) than in controls (*p* < 0.0001).

In CAYAs, the leading cardiovascular causes of death were cerebrovascular diseases (28.1%), arrhythmia (22.9%), heart failure (17.1%), and IHD (15.0%), and in controls, the leading cardiovascular causes of death were IHD (28.8%), arrhythmia (22.2%), and cerebrovascular diseases (18.0%).

### Mortality and sociodemographic factors in CAYAs

After adjusting for sex, age, demographic and socioeconomic factors, male CAYAs had a 1.94-fold greater risk of all-cause mortality after their first CVD than female CAYAs did (HR 95% CI 1.74–2.17, *p* < 0.0001), and a 1.65-fold greater risk for male controls was observed (HR 95% CI 1.52–1.79, *p* < 0.0001). In pediatric patients receiving the standard protocol, there was no significant difference in mortality between females and males (HR 1.03, 95% CI 0.92–1.16, *p* = 0.56), although a higher risk was observed for males with CNS malignancies (HR 1.26, 95% CI 1.06–1.50, *p* = 0.008).

CAYAs residing in the central part of Sweden presented a 1.20-fold (HR 95% CI 1.06–1.36, *p* < 0.0040) greater risk of all-cause mortality than did CAYAs living in the south. Median municipal income, living in an urban or rural area, or distance to a hospital did not influence the risk of all-cause mortality in CAYAs.

In contrast, CAYAs who had a higher level of education (HR 0.19 95% CI 0.16–0.22, *p* < 0.0001) or who were married (HR 0.44 95% CI 0.39–0.49, *p* < 0.0001) presented a lower all-cause mortality risk (Supplementary data Tables 3 and 4). For those who survived beyond the age of 30, these findings remained consistent.

## Discussion

This comprehensive study, which spans six decades of CVD in all young cancer patients in Sweden (with free access to health care), revealed that the onset of CVD and the occurrence of multiple CVDs consistently appeared earlier and at a younger age in CAYAs than in controls. Compared with controls, CAYAs with CVD had a higher mortality rate and died at a younger age. Additionally, the study demonstrated that sociodemographic factors were associated with a greater risk of mortality in CAYAs than in matched controls.

### Cardiovascular risk factors and CVD in CAYAs

Cancer and CVD are linked through shared risk factors such as smoking, obesity, poor diet, and aging [12–15]. Inflammation, oxidative stress, and genetic factors contribute to both conditions, [16–18] whereas cancer treatments such as chemotherapy and radiation can damage the heart and blood vessels [19, 20]. Many reports have established that a higher incidence of CVRF at a young age increases the risk of CVD. Owing to various risk factors, cancer treatment alone may not be sufficient to determine the risk of CVD later in life. In the present study, the overall burden of CVRFs was greater than that of controls despite the younger age of CAYAs at study completion. However, the incidence of IHD was lower in CAYAs, which may be attributed to their younger age at the end of the study or the distribution of various CVDs within the groups. Many patients with IHD in Sweden (68%) had a history of ever smoking [12] and these numbers align with the current study, which revealed that 65.4% of CAYAs were ever smokers. According to the Public Health Agency of Sweden, the number of individuals who smoke daily has decreased over the years. Nevertheless, disparities exist among different groups and are influenced by factors such as education, income level, and employment status. Compared with the general population, cancer survivors are at a greater risk of developing IHD, partly because of CVRFs [16].

According to the European Society of Cardiology (ESC) guidelines, risk assessment is recommended for individuals with elevated blood pressure to identify those at high risk for other CVD [21]. Hypertension increases the risk for subsequent CVD and may potentiate therapy-associated risks for major events [22]. We found that hypertension was the most common incident CVD; approximately 40% of CAYAs and controls developed hypertension, which aligns with previous findings by Kooijmans et al. [23]. In the ALiCCS study, childhood cancer survivors were at a markedly increased risk for CVD throughout their lives, with hypertension being twice as common at hospital admission [6]. Hypertension was also the most frequent CVD among CAYAs with two or more incident CVDs, suggesting that hypertension follow-up should be prioritized for young cancer survivors.

The prevalence of diabetes amongst CAYAs in the current study doubled compared with that in similar age groups in the general population, which was not observed in the control group. Smoking and diabetes could accelerate the development of CVD in CAYAs, increasing their risk at a younger age.

### High prevalence of cervical cancer in CAYAs with incident CVD

The distribution of index malignancies was similar to that reported in other international registry studies, except for the high number of young females with cervical cancer, including high-grade squamous intraepithelial lesions, which has not been included in previous cardiovascular reports from comparable populations [3, 6, 9]. Human papillomavirus (HPV) infection is associated with cervical cancer, and survivors of cancer are known to have a greater risk of HPV-associated secondary cancers and CVD [24]. This may be partly attributed to the previously identified correlation between having multiple sexual partners and higher rates of smoking and alcohol consumption, which in turn heighten the risk of CVD. Individuals with obesity and those with metabolic syndrome who have HPV infection are at an increased risk for CVD. Therefore, CAYAs with a history of cervical cancer represent an important group for future cardiovascular studies [25].

### Increased risk of all-cause and cardiovascular mortality in CAYAs

The risk of cardiovascular mortality is elevated following cancer and its treatment in young individuals [26, 27]. This pattern was also observed in the current study, where CAYAs presented 2.17-fold and 2.43-fold higher risks of cardiovascular mortality and all-cause mortality, respectively, than controls did. The incidence of underlying causes of cardiovascular mortality is also in line with the findings of Bottinor et al. [10], who demonstrated that after major cardiovascular events such as heart failure, IHD or cerebrovascular disease, survivors face higher all-cause mortality risks than their siblings do.

### Mortality and sociodemographic factors in CAYAs

Sociodemographic and modifiable risk factors increase the likelihood of CVD in young cancer survivors, sometimes disproportionately compared with controls [4]. While males typically have a greater risk for CVD earlier in life than females do, the current study, which includes a substantial number of female participants with locally treated cervical cancer, suggests that over time, the mortality risk among young females with cancer has become comparable to that of males in the control group. Among children who received cancer treatment, the risk of mortality after CVD was similar for females and males, except that male with a history of childhood CNS malignancies had an increased risk.

Having a background from outside Sweden for controls was linked with higher mortality. Long distances to a hospital may increase mortality in both CVD and cancer patients [28, 29]. Most people live in central and southern urban areas, including municipalities with the best education, healthcare, and transportation access. In the present study, living in central regions was associated with an increased mortality risk, but neither the distance to the nearest hospital nor the municipality's wealth impacted mortality for CAYAs.

Healthcare and sociodemographic factors impact the education and career challenges of cancer survivors. Studies indicate that cancer survivors often face disruptions in their education and career plans, leading to fewer individuals obtaining college or university degrees [30]. Berkman et al. [4] reported no evidence indicating that the likelihood of CVD varied on the basis of educational attainment between AYA survivors and controls. In contrast to the current study, although CAYAs completed primary school, continuing their education at higher levels appeared more challenging for them than for the controls. However, when CAYAs graduated from a university education, there was a greater reduction in mortality risk than in controls. Civil status can reflect adaptation in adulthood. In Sweden, cohabitation is as common as marriage, but it is not officially recorded. In the present study, CAYAs were married less frequently than controls were, and marriage positively impacted mortality rates. Differences in education and civil status beyond the age of 30 were also linked to mortality, suggesting that the mortality rate in childhood does not fully explain the findings.

## Strengths and limitations

This study has strong internal validity because high-quality national patient registers with low dropout rates cover decades of evolving diagnostic criteria and screening practices for cancer and CVD. However, register studies often encounter selection, confounding, measurement, and reporting biases. It is crucial to note limitations from data spanning different periods, which may not completely overlap, and the inclusion of individuals who died before certain registers were established, affecting conclusions, generalizability, and comprehensiveness.

Variations in diagnostic criteria, treatment practices, and healthcare access over the decades can introduce confounding effects that impact the reliability of the findings. Additionally, the ICD system has undergone multiple revisions, resulting in potential disease classification and staging inconsistencies across different periods.

The study included treatment information for children with leukemia, CNS malignancies, lymphoma, and cancer of the testis. In this study, most female patients did not receive systemic cancer treatment; therefore, sex-specific comparisons are more accurate in the sub analysis of the cancer types listed above. While the current study offers valuable insights, the absence of individual treatment data for other cancers affects its comprehensiveness. However, cancer treatment has advanced, especially in terms of protecting the heart during chest radiation, and the introduction of anthracycline treatment has improved survival rates and increased the likelihood of living long enough to develop CVD. Additionally, reductions in smoking, hypercholesterolemia, and the treatment of other CVRFs among the population, along with enhanced cardiovascular care, have had a substantial impact since the beginning of the study.

Moreover, survival rates for both cancer patients and CVD patients have improved significantly because of advancements in early detection, surgical and intervention techniques, pharmacological interventions, and preventive measures. In Sweden, childhood cancer survivors now receive nationwide, guideline-based long-term follow-up care aimed at the early detection of late effects. Until recently, this follow-up was inconsistent and primarily focused on issues other than cardiovascular risk factors and diseases. There is no follow-up beyond the regular 5-year oncological follow-up for young adults, except in cases of certain cancers treated with advanced therapies such as stem cell transplantation.

These temporal changes introduce a potential systematic bias when analyzing long-term outcomes, as earlier-diagnosed patients experienced vastly different prognostic factors and treatment paradigms than those diagnosed in later cohorts did.

However, this study provides a foundational framework. It serves as a basis for constructing a large database, enabling more in-depth studies to gain a deeper understanding of different CVDs and their timing after cancer. Future research should aim to develop interventions that address young cancer survivors’ overall well-being and social integration.

The results align with those of other European and US studies, and it is essential to compare entire populations across countries, considering that access to general healthcare and cardiovascular care varies widely. With aligned results, comparable studies can be conducted, and data can be combined into larger registry studies.

## Conclusions

This comprehensive study on the timing of cardiovascular morbidity, mortality, and the impact of sociodemographic factors in young cancer patients indicates that these patients develop CVD earlier in life and face a higher mortality rate. Age, male sex, and living in the central of Sweden were linked to increased mortality after the first CVD. Compared with controls, CAYAs also marry less frequently and have lower education levels. In adulthood, marriage provided benefits, whereas higher education was associated with a greater reduction in mortality among CAYAs than among controls. These factors interconnect, influencing future quality of life and outcomes, many of which can be modified through appropriate screening strategies and timely interventions.

## Disclosures

EH is a co-founder and board member of the MedTech company TrueDose AB, which produces at-home blood sampling kits and has received speaker and consultancy fees from Bristol-Myers Squibb, Pfizer, and Amgen, all paid to Karolinska University Hospital.

KRW reports unrelated speaker and consultancy fees from Roche, Pfizer, Organon, Ibsa, Merck and Ferring Pharmaceuticals and unrelated grants from Novo Nordisk and Ferring.

PK reports a fee for a lecture from AstraZeneca and Boehringer Ingelheim and is a member of the Advisory Board from Pharmacosmos, Novartis and AstraZeneca. LH reports unrelated modest consultation fees from Astellas and Novartis. The remaining authors have nothing to disclose.

## Supplementary Information

Supplementary Material 1.

## Data Availability

No datasets were generated or analysed during the current study.

## Abbreviations

AYA: Adolescent and young adults
CAYA: Children, adolescents, and young adults
CNS: Central nervous system
CVD: Cardiovascular disease
ICD-10: International Classification of Diseases 10th revision
IHD: Ischemic heart disease
