# Supplementary material for: Timing and combinations of cardiovascular diseases in survivors of childhood, adolescent, and young adulthood cancer
Source: Cardiooncology. 2025 Oct 17;11:92. doi: 10.1186/s40959-025-00385-8 (PMC12532896; doi:10.1186/s40959-025-00385-8)
Supplement: Supplementary file 1 — Supplementary Material 1. [file 40959_2025_385_MOESM1_ESM.pdf]

Supplemental Appendix.

Timing and Combinations of Cardiovascular Diseases in Survivors of Childhood, Adolescence, and Young Adulthood cancer

**Supplemental Figures**	Page
**Figure 1** Flowchart	2
**Figure 2.** National Registers over Time	2
**Figure 3.** All-cause mortality over time, divided in decades of index	7
**Figure 4.** Cardiovascular mortality over time-divided in decades of index	8
**Figure 5.** Mortality over time-divided into age groups at index	9
**Figure 6.** Cumulative all-cause-and cardiovascular mortality by index cancer diagnosis	10
**Supplemental Tables**	
**Table 1**. Swedish National Registers used to obtain data	3
**Table 2**. ICD 10 codes	4
**Table 3.** Sociodemographic factors and covariates; association with all-cause mortality	5
**Table 4.** Sociodemographic factors, association with all-cause mortality. Adjusted	6
**Table 5 a, b and c.** CVDs in children <18 years (total(a), female(b) and males(c)) with Leukemia, CNS malignancies, Lymphoma, and Testis cancer that received treatment according to standard protocols	11
**Table 6.** All cause and cardiovascular mortality outcomes after the first cardiovascular disease in children <18 years with Leukemia, CNS malignancies, Lymphoma, and Testis cancer that received treatment according to standard protocols.	14
**Table 7.** Baseline Sociodemographic factors for females and males.	15

Figure 1. Flowchart of the study selection process
Flowchart illustrates the selection process. A total of 2,571 cancer patients were excluded because they were diagnosed with cancer prior to 1968 and were not included in the Statistics Sweden registry. Additionally, 2 patients were unmatched for unspecified reasons, and 32 had fewer than 5 eligible controls available. Matching 1:5 based on age, sex and living area at index. Abbreviations: CVD=cardiovascular disease (ICD-10 I00-I99, and G45).
CAYAs= Children, adolescents, and young adults with cancer (<25 years of age).

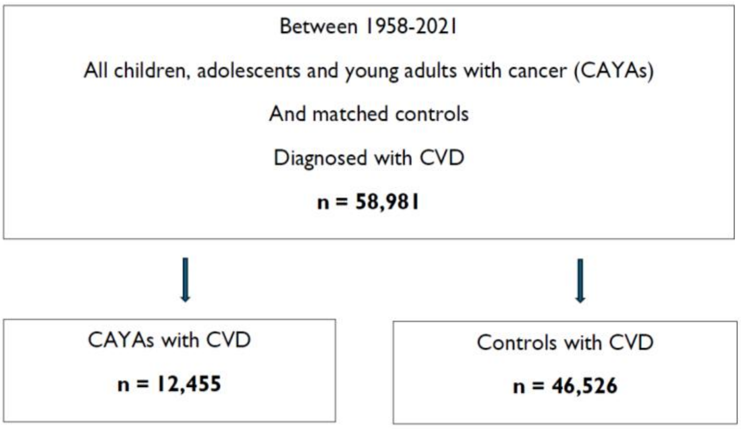

Figure 2. Swedish National Registers used in this study, and their timeframe.

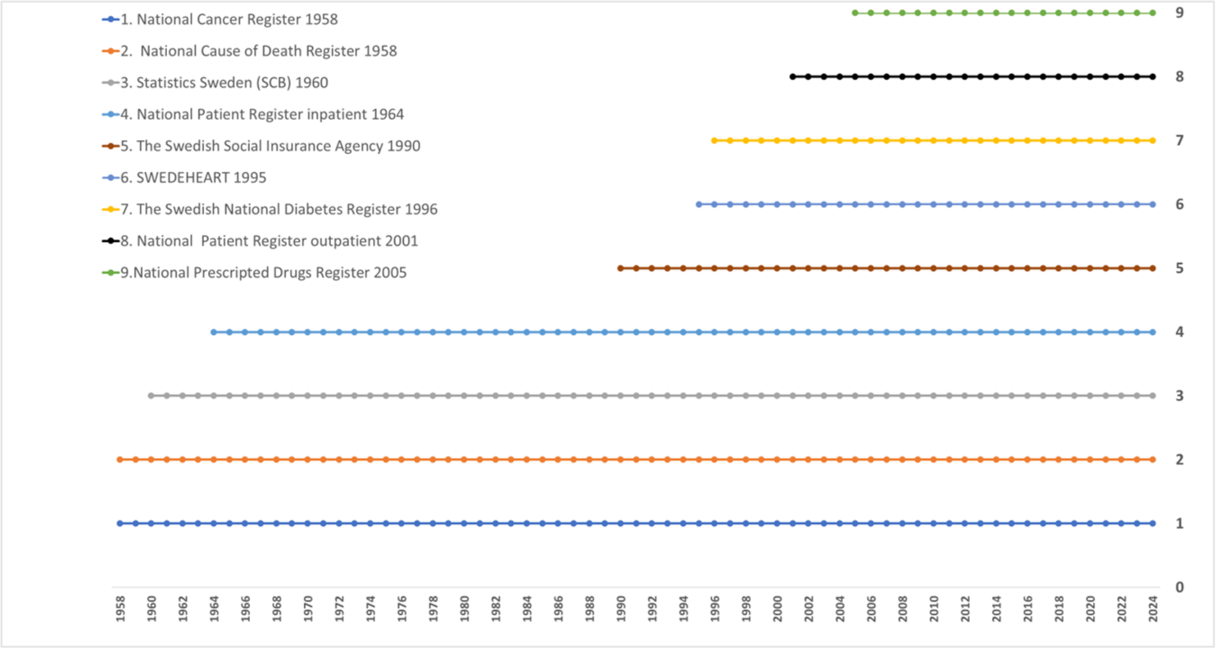

Table 1. Swedish National Registers used to obtain data.

**Register**[1]	**Information**	**Information retrieved**
National Cancer Register<br>1958	Started in 1958 and includes data on histological type-site-date of diagnosis-date and cause of death-with a coverage rate of 96%. All tumors in the register are coded and registered according to ICD-7 and ICD10.[2,3]	Index cancer diagnosis and date
The Cause of Death Register<br>1952	The Swedish Cause of Death Register is a high quality virtually complete register of all deaths in Sweden since 1952.[4]	ICD code for the main cause of death and date of death.
Statistics Sweden<br>1960	Statistics Sweden (SCB) is responsible for official statistics and for other government statistics since 1960.<br>SCB also provides a Longitudinal integrated database for health insurance and labor market studies (LISA)	Matched controls1:5<br>Residence at index diagnosis<br>Municipality number at index diagnosis median income in 2021 with Gini-coefficient (0-1 where higher values indicate greater inequality),-civil status (latest registered unmarried or married)-sex (at index) and education levels (latest registered completed elementary school, upper secondary school, or university/postgraduate education).
The Swedish Social Insurance Agency<br>1990	Conducts supporting research in the field of social insurance. Investigates and decides on the right to compensation from social insurance. It includes, amongst other things, compensation for sickness benefit.	Days sick-leave and disability pension
National Patient Register.<br>1964	Captures data on hospitalized patients (inpatients) and those who have received medical care from healthcare providers without hospital admission (outpatients) for diagnosis-treatment-or follow-up. The inpatient register has been operational since 1964-while the outpatient register was started in 2001. These registers are essential to healthcare information infrastructure-monitoring-and improving healthcare services nationwide.[5]	Diagnosis after index date and days in hospital care. Additional diagnoses were collected from the outpatient register.
National Prescribed Drug Register<br>2005	The register contains information of all prescribed drugs dispensed in pharmacies since 2005.	Use of lipid lowering drugs.
The Swedish National Diabetes Register<br>1996	The Swedish National Diabetes Register (NDR) was formed in 1996. It's a quality register including patients with both type 1 and type 2 diabetes.	Tobacco use. BMI (body mass index, BMI ≥ 30 kg/m²).
SWEDEHEART<br>(Swedish Web system for Enhancement and Development of Evidence-based care in Heart disease Evaluated According to Recommended Therapies)<br>2009	SWEDEHEART includes several registries. RIKS-HIA-the Swedish register of cardiac intensive care (1991) and a national quality register since 1995. By 2008 all hospitals in Sweden, Iceland and Aland that provided acute cardiac care are included.<br>SEPHIA is a register of secondary prevention after myocardial infarction.<br>The Swedish Angiography and Angioplasty Registry-SCAAR (1998)- register coronary angiographies and percutaneous interventions.<br>The Swedish Heart Surgery Register (1992) covers all 8 centers of cardiac surgery in Sweden.<br>The Swedish Heart Failure Registry-SwedeHF (2003) and evaluate the are of heart failure nationwide.	Tobacco use. BMI.

References:

1. Ludvigsson JF-Almqvist C-Bonamy AK-Ljung R-Michaelsson K-Neovius M-et al. Registers of the Swedish total population and their use in medical research. Eur J Epidemiol. 2016;31(2):125-36.
2. Socialstyrelsen. Swedish National Cancer Register. Available from: https://www.socialstyrelsen.se/en/statistics-and-data/registers/register-information/swedish-cancer-register/.
3. Barlow L-Westergren K-Holmberg L-Talback M. The completeness of the Swedish Cancer Register: a sample survey for year 1998. Acta Oncol. 2009;48(1):27-33.
4. Brooke HL-Talback M-Hornblad J-Johansson LA-Ludvigsson JF-Druid H-et al. The Swedish cause of death register. Eur J Epidemiol. 2017;32(9):765-73.
5. Ludvigsson JF-Andersson E-Ekbom A-Feychting M-Kim JL-Reuterwall C-et al. External review and validation of the Swedish national inpatient register. BMC Public Health. 2011;11:450.
6. Ljung R-Lundgren F-Appelquist M-Cederlund A. The Swedish dental health register-validation study of remaining and intact teeth. BMC Oral Health. 2019;19(1):116.

Table 2. ICD 7-9 codes from 1958-1997 were translated into ICD-10 codes.

**Cardiovascular diseases**	**I00-I99**
Rheumatic heart disease	I00-I09
Hypertension	I10-I15
Coronary artery diseases	I20-I25
Pulmonary heart diseases	I26-I28
Myo-endo-and pericarditis	I30-I33-I38-I41
Valvular diseases	I34-I37
Arrhythmias	I44-I49
Heart failure and cardiomyopathy	I50-I42-I43
Cerebrovascular	I60-I69
Diseases of arteries-arterioles and capillaries	I70-I79
Vein diseases	I80-I87
Other CVD	I51-I52, I88-I89, I95-I99
**Other**	
Chronic lower respiratory disease	J44
Interstitial lung disease-including fibrosis	J84
Diabetes mellitus	E10-E11
Lipidaemia	E78
Chronic kidney disease	N18
Hypothyroidism	E03
Hyperthyroidism	E05

The translation to ICD-10 was based on the recommendations for mapping from ICD-9 to ICD-10. The electronic supplementary material for converting between ICD 7-10 is available from the National Board of Health and Welfare, and then manual matched by medical professionals.

**Table 3.** Age, sex and sociodemographic factors and covariates; association with all-cause mortality (univariable).						
	**CAYAs**			**Controls**		
	**HR**	**95% CI**	p-value	**HR**	**95% CI**	p-value
Male	2.20	2.04-2.38	<0.0001	1.64	1.54-1.74	<0.0001
Age at index	0.96	0.95-0.96	<0.001	1.02	1.02-1.03	<0.0001
Area of birth						
Sweden	*ref*			*ref*		
Europe	0.87	0.72-1.06	0.17	1.21	1.08-1.35	<0.0001
Other	1.19	0.90-1.56	0.22	0.49	0.35-0.68	<0.0001
**Part of Sweden at index**						
South	*ref*			*ref*		
Central	1.34	1.24-1.46	<0.0001	1.16	1.09-1.24	<0.0001
North	1.29	1.14-1.46	<0.0001	1.17	1.06-1.28	0.001
**Inhabitant/km2 in municipal. no**	1.00	1.00-1.00	0.005	1.00	1.00-1.00	0.0003
≥ 2241	*ref*			*ref*		
15-2241	0.85	0.76-0.95	0.003	0.82	0.76-0.89	<0.0001
<15	1.04	0.89-1.21	0.63	0.93	0.83-1.04	0.21
**Median income in municipal. SEK**	1.00	1.00-1.00	0.22	1.00	1.00-1.00	0.27
> 363 000	*ref*			*ref*		
285 000-363 000	0.83	0.74-0.92	0.0003	0.96	0.88-1.05	0.38
< 285 000	0.89	0.76-1.03	0.13	1.01	0.89-1.13	0.94
**Gini coefficient 0-1**	0.62	0.25-1.55	0.31	1.13	0.56-2.29	0.74
<0.31	*ref*			*ref*		
0.31-0.42	0.84	0.75-0.95	0.006	0.92	0.83-1.01	0.079
>0.42	0.99	0.84-1.17	0.92	0.96	0.84-1.10	0.56
**Proximity to hospital. km**	1.00	1.00-1.00	0.026	1.00	1.00-1.00	0.36
<30	*ref*			*ref*		
30-100	1.09	0.99-1.20	0.096	0.97	0.90-1.05	0.479
> 100	1.20	0.74-1.97	0.46	1.61	1.19-2.20	0.002
**Highest education level**						
Elementary school	*ref*			*ref*		
Upper secondary school	0.41	0.38-0.45	<0.0001	0.44	0.42-0.47	<0.0001
University and postgraduate	0.19	0.17-0.22	<0.0001	0.21	0.19-0.23	<0.0001
**Civil status**						
Unmarried	*ref*			*ref*		
Married or reg· Partner	0.28	0.25-0.31	<0.0001	0.48	0.44-0.51	<0.0001
**Sick leave, days**						
Sick-leave >180 days	0.54	0.50-0.59	<0.0001	0.97	0.91-1.03	0.30

**Table 4.** Sex and sociodemographic factors association with all-cause mortality. Adjusted for 9 factors (multivariable).						
	**CAYA adjusted**			**Controls adjusted**		
	**HR**	**95% CI**	p-value	**HR**	**95% CI**	p-value
Male	1.94	1.74-2.17	<0.0001	1.65	1.52-1.79	<0.0001
Age at index	1.05	1.04-1.06	<0.0001	1.05	1.04-1.05	<0.0001
**Area of birth**						
Sweden	ref					
Europe	0.84	0.62-1.14	0.27	1.20	1.02-1.40	0.026
Other	0.79	0.53-1.18	0.25	0.45	0.28-0.71	0.0007
**Part of Sweden at index**						
South	ref					
Central	1.20	1.06-1.36	0.004	1.14	1.04-1.25	0.004
North	1.18	0.98-1.42	0.082	1.15	1.01-1.32	0.037
**Inhabitant/km2 in municipal. no**						
≥ 2241	*ref*					
15-2241	0.89	0.73-1.09	0.27	0.75	0.65-0.86	<0.0001
<15	0.89	0.65-1.21	0.46	0.84	0.67-1.04	0.110
**Median income in municipal. SEK**						
> 363 000	*ref*					
285 000-363 000	1.10	0.88-1.39	0.39	1.08	0.91-1.28	0.38
< 285 000	1.04	0.78-1.39	0.79	1.02	0.82-1.27	0.85
**Gini coefficient 0-1**						
<0.31	*ref*					
0.31-0.42	0.98	0.81-1.19	0.84	1.01	0.88-1.17	0.85
>0.42	1.03	0.74-1.45	0.85	0.89	0.69-1.14	0.35
**Proximity to hospital. km**						
<30	*ref*					
30-100	0.96	0.82-1.12	0.60	0.94	0.84-1.06	0.32
> 100	0.90	0.42-1.94	0.80	1.59	1.09-2.30	0.015
**Highest education level**						
Elementary school	*ref*					
Upper secondary school	0.38	0.34-0.43	<0.0001	0.44	0.40-0.48	<0.0001
University and postgraduate	0.19	0.16-0.22	<0.0001	0.21	0.19-0.24	<0.0001
**Civil status**						
Unmarried	*ref*					
Married or reg· Partner	0.44	0.39-0.49	<0.0001	0.52	0.49-0.57	<0.0001
**Sick leave days**						
Sick-leave >180 days	0.82	0.73-0.91	0.0003	0.95	0.88-1.03	0.20

Figure 3. All-cause mortality over time, divided into decades of index. CAYAs in panel (A) and Controls in panel (B).

A

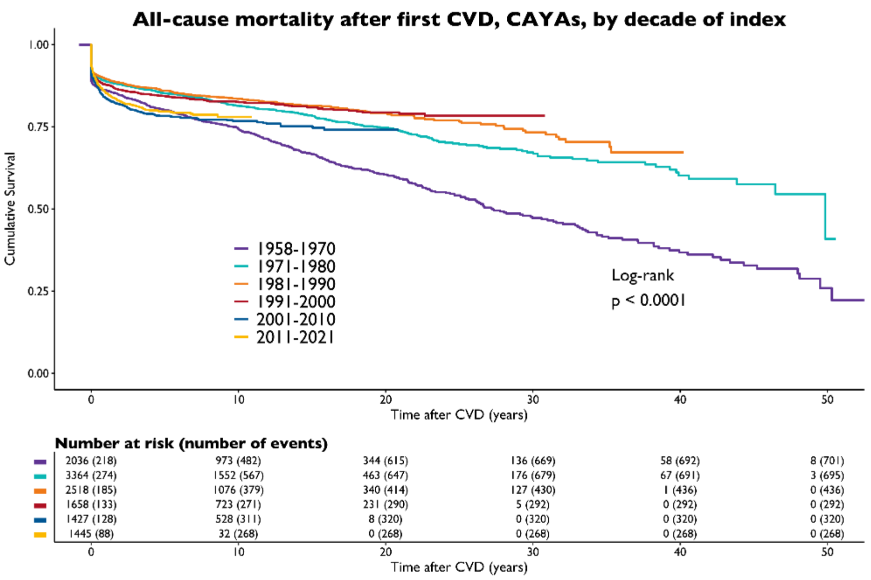

B

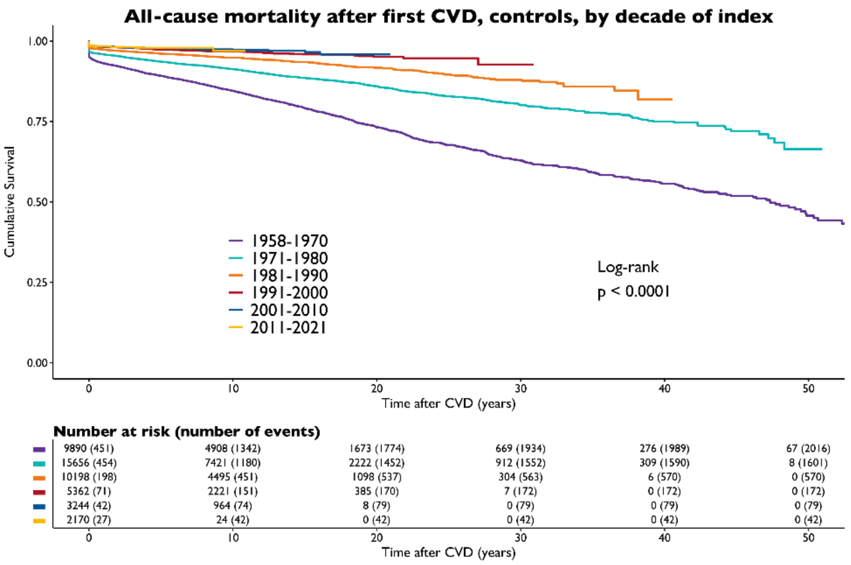

Figure 4 Cardiovascular mortality over time-divided in decades of index. CAYAs in panel (A) and Controls in panel (B).

A

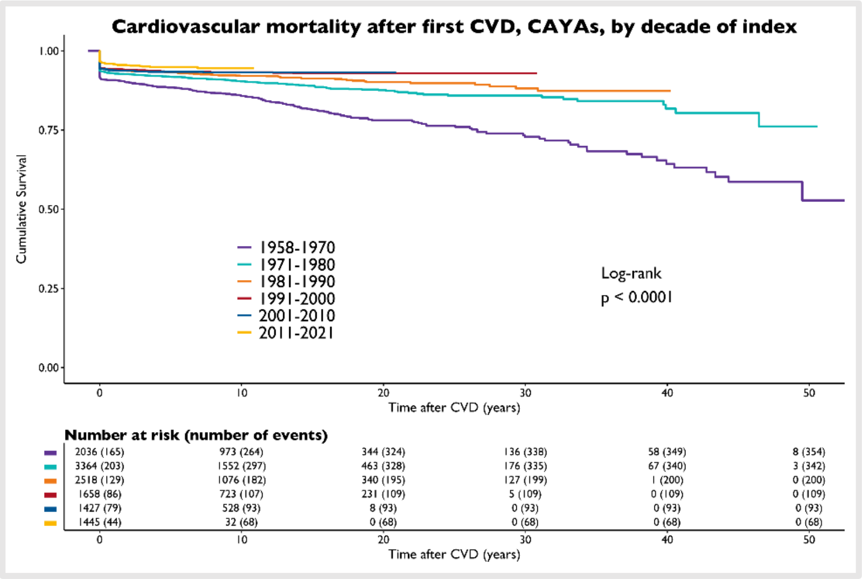

B

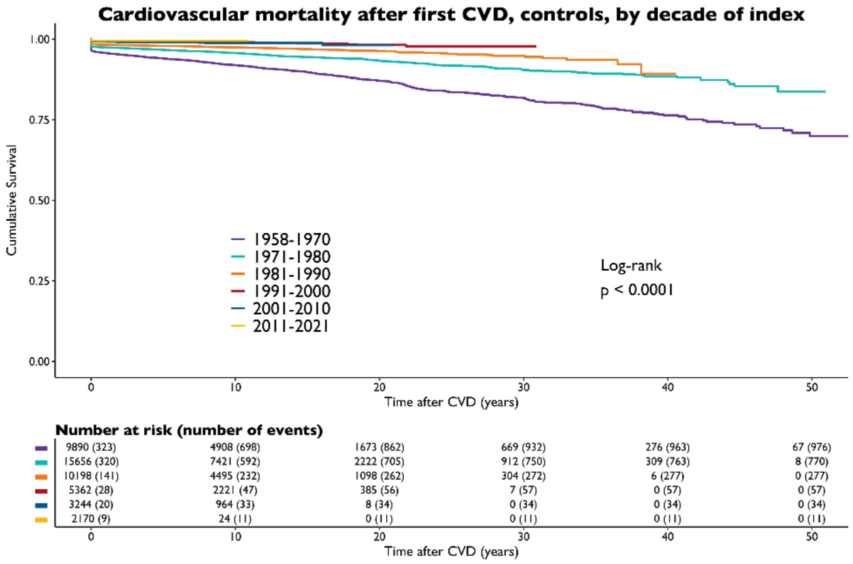

Figure 5. Mortality over time-divided into age groups at index. All-cause mortality in panel (A) and Cardiovascular mortality in panel (B).

A

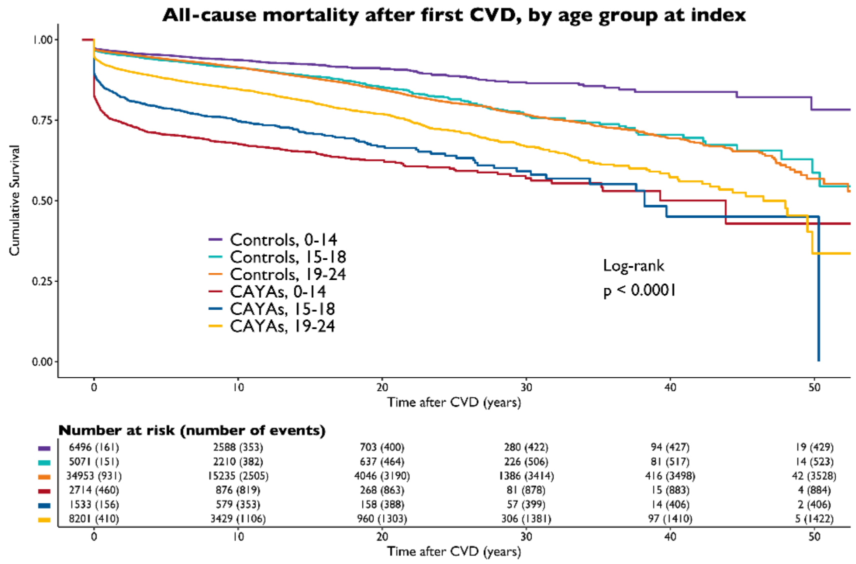

B

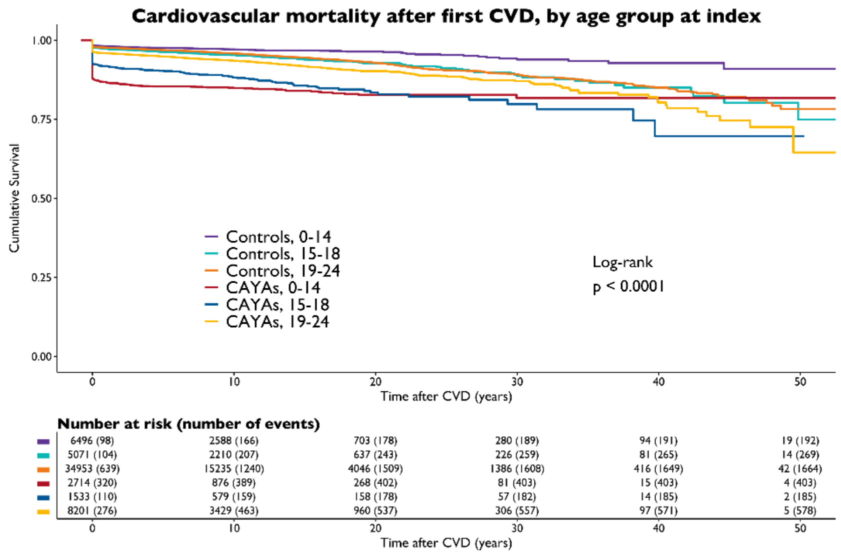

Figure 6. Cumulative all-cause-and cardiovascular mortality by index cancer diagnosis

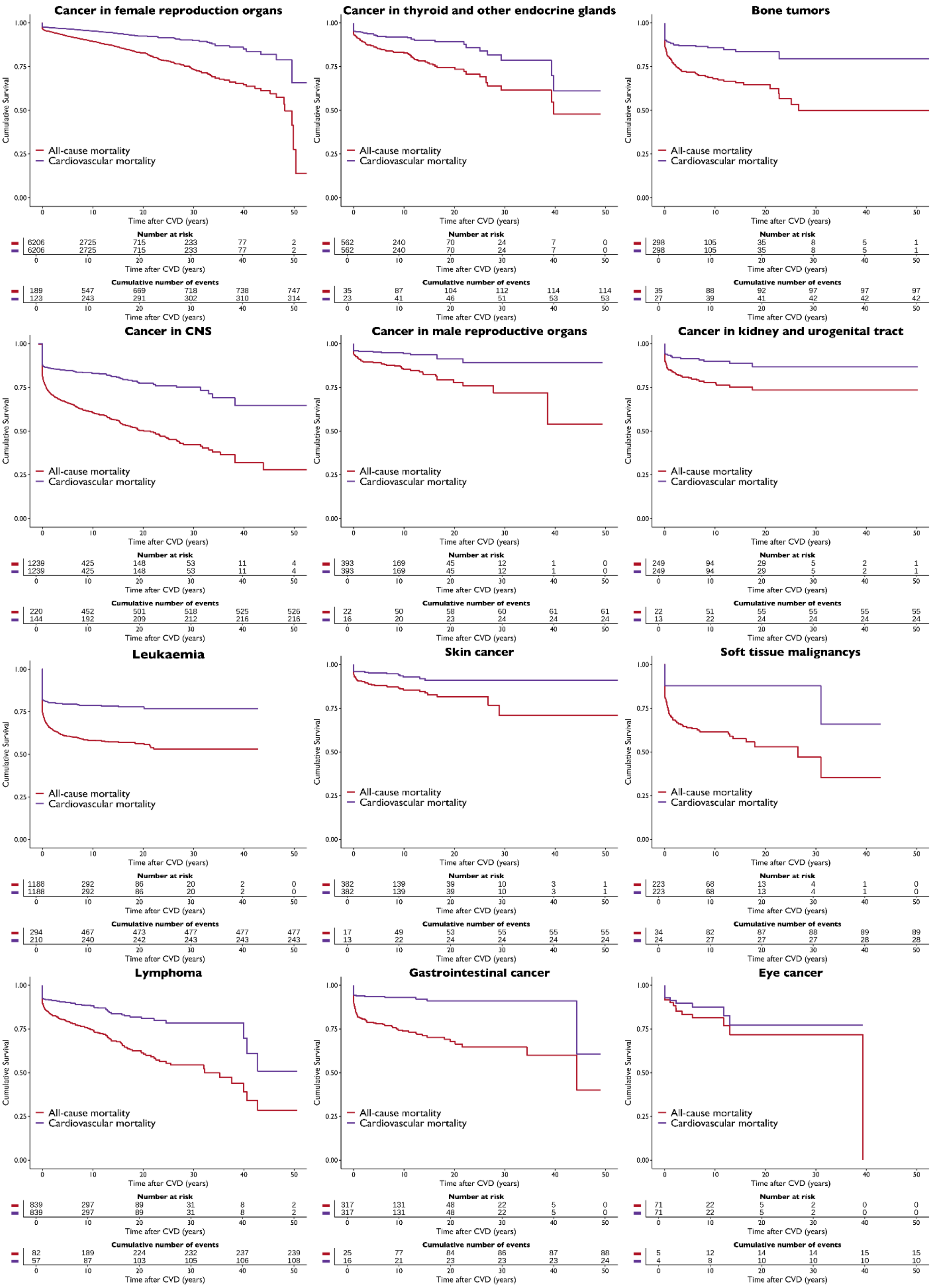

Table 5a. Cardiovascular diseases (CVD) after index in children <18 years with Leukemia, CNS malignancies, Lymphoma, and Testis cancer that received treatment according to standard protocols.

	CNS tumors n = 1242	Leukemia n = 1189	Lymphoma n = 839	Testis cancer n = 362
Age at index median (IQR)	13 (6-19)	8 (3-16)	18 (14-22)	22 (19-23)
Time from index to CVD years median (IQR)	15.2 (1.6-31.2)	1.2 (0.2-8.1)	10.7 (0.5-26.6)	14.1 (1.0, 27.6)
Median age at CVD years median (IQR)	27.7 (16.0-44.6)	14.3 (6.4-23.1)	27.9 (19.2-44.4)	35.3 (23.3, 47.9)
**Cancer treatment** n (%)	1207.0 (97.2)	1055.0 (88.7)	678.0 (80.8)	362.0 (100.0)
Radiotherapy	1087 (90.1)	298.0 (28.2)	348 (51.3)	243 (67.1)
Anthracycline	0 (0)	1021 (96.8)	607 (89.5)	0 (0)
Other drugs	1000 (82.9)	1055 (100)	375 (55.3)	276 (76.2)
**Cardiovascular diseases** n (%) **Time to CVD and Age at CVD** years median (IQR)				
Hypertension (HTN)	298 (24.0)	296 (24.9)	215 (25.6)	137 (37.8)
Time to HTN	33.5 (20.2-44.9)	1.4 (0.2-19.2)	29.5 (16.0-38.1)	29.2 (19.1-38.3)
Age at HTN	47.9 (32.7-58.9)	12.4 (4.4-29.2)	46.3 (33.4-55.6)	48.0 (40.0-57.3)
Arrhythmias (ARY)	223 (18.0)	165 (13.9)	200 (23.8)	82 (22.7)
Time to ARY	16.4 (2.0-33.2)	2.4 (0.4-18.1)	27.5 (12.6-36.7)	23.1 (10.9-36.2)
Age at ARY	27.7 (13.0-49.0)	16.5 (8.0-26.7)	45.5 (30.7-56.3)	44.4 (32.7-55.4)
Ischemic heart diseases (IHD)	86 (6.9)	40 (3.4)	159 (19.0)	44 (12.2)
Time to IHD	38.5 (25.5-46.8)	17.1 (0.6-34.6)	28.6 (21.7-34.9)	28.7 (20.2-38.3)
Age at IHD	53.3 (43.5-64.4)	33.8 (7.9-48.5)	47.3 (40.2-54.7)	50.5 (44.2-61.1)
Heart failure (HF)	99 (8.0)	181 (15.2)	178 (21.2)	30 (8.3)
Time to HF	27.4 (8.0-43.2)	2.9 (0.5-14.0)	26.6 (7.8-36.0)	31.0 (15.3-37.7)
Age at HF	39.0 (21.7-57.9)	16.1 (7.8-25.6)	44.0 (24.6-54.6)	51.1 (39.3-57.0)
Cerebrovascular diseases	512 (41.2)	285.0 (24.0)	92.0 (11.0)	39 (10.8)
Time to Cerebrovascular diseases	14.2 (0.7-30.8)	0.9 (0.1-3.6)	24.6 (4.6-36.5)	28.0 (16.6-37.7)
Age at Cerebrovascular diseases	27.3 (15.4-44.5)	14.9 (7.6-22.2)	43.4 (22.8-56.1)	48.2 (35.7-58.9)
Intracranial hemorrhage (ICH)	262 (21.1)	195 (16.4)	27 (3.2)	10 (2.8)
Time to ICH	7.3 (0.2-26.9)	0.7 (0.1-2.1)	13.3 (0.5-33.2)	12.1 (0.8-24.3)
Age at ICH	23.1 (13.0-42.6)	14.0 (6.8-20.1)	32.3 (16.4-45.1)	33.1 (23.3-41.3)
Cerebral infarction (CVA)	167 (13.4)	40 (3.4)	41 (4.9)	15 (4.1)
Time to CVA	27.7 (9.8-38.5)	7.1 (0.2-28.2)	25.5 (8.5-39.3)	35.3 (25.2-46.8)
Age at CVA	41.2 (24.3-53.2)	20.7 (8.3-42.1)	44.9 (27.5-58.5)	53.5 (48.2-63.8)

Table 5b. Cardiovascular disease (CVD) after index in **female** children <18 years with Leukemia, CNS malignancies, Lymphoma, and Testis cancer that received treatment according to standard protocols.

	CNS tumors n = 541	Leukemia n = 492	Lymphoma n = 306	Testis cancer n = 0
Age at index median (IQR)	13.0 (6.0-19.0)	7.0 (3.0-15.0)	18.0 (15.0-21.0)	..
Time from index to CVD years median (IQR)	15.4 (1.8-31.0)	1.6 (0.2-9.0)	8.9 (0.5-24.3)	..
Median age at CVD years median (IQR)	27.2 (16.0-44.3)	14.1 (5.7-23.3)	26.6 (19.0-42.2)	..
**Cancer treatment** n (%)	528 (97.6)	438 (89.0)	254 (83.0)	..
Radiotherapy	475 (90.0)	139 (31.7)	123 (48.4)	..
Anthracycline	0 (0)	422 (96.3)	226 (89.0)	..
Other drugs	432 (81.8)	438 (100.0)	160 (63.0)	..
**Cardiovascular diseases** n (%) **Time to CVD and Age at CVD** years median (IQR)				
Hypertension (HTN)	118 (21.8)	112 (22.8)	70 (22.9)	..
Time to HTN	34.0 (19.7-47.2)	1.0 (0.2-25.0)	25.6 (14.1-35.9)	..
Age at HTN	49.1 (30.8-62.1)	12.5 (5.2-33.9)	42. (29.5-55.6)	..
Arrhythmias (ARY)	96 (17.7)	67 (13.6)	74 (24.2)	..
Time to ARY	11.6 (2.0-27.3)	1.8 (0.2-21.6)	22.3 (5.6-31.2)	..
Age at ARY	24.4 (12.2-40.6)	12.4 (3.3-25.7)	39.0 (24.1-54.1)	..
Ischemic heart diseases (IHD)	26 (4.8)	13 (2.6)	39 (12.7)	..
Time to IHD	34.9 (17.2-46.7)	2.4 (0.7-38.0)	27.5 (22.2-34.7)	..
Age at IHD	52.4 (28.8-64.4)	25.1 (9.4-53.0)	47.1 (38.1-54.9)	..
Heart failure (HF)	38 (7.0)	76 (15.4)	61 (19.9)	..
Time to HF	29.1 (10.2-43.3)	3.8 (0.7-11.8)	26.7 (11.9-32.6)	..
Age at HF	40.9 (22.8-57.7)	15.0 (6.7-26.0)	45.5 (25.7-54.0)	..
Cerebrovascular diseases	216 (39.9)	111 (22.6)	29 (9.5)	..
Time to Cerebrovascular diseases	12.4 (0.7-28.5)	0.7 (0.1-3.4)	12.7 (0.6-32.8)	..
Age at Cerebrovascular diseases	25 (15.4-42.5)	14 (7.1-21.5)	32 (17.1-54.9)	..
Intracranial hemorrhage (ICH)	99.0 (18.3)	74.0 (15.0)	6.0 (2.0)	..
Time to ICH	4.5 (0.2-25.6)	0.5 (0.1-2.0)	8.0 (0.2-13.3)	..
Age at ICH	20.8 (10.6-41.9)	13.0 (5.5-19.3)	27.2 (14.2-33.6)	..
Cerebral infarction (CVA)	65 (12.0)	16 (3.3)	11 (3.6)	..
Time to CVA	24.8 (7.9-38.1)	0.3 (0.1-7.8)	7.5 (0.5-40.6)	..
Age at CVA	34.8 (19.1-50.2)	11.1 (4.4-25.6)	23.5 (16.1-63.6)	..

Table 5c. Cardiovascular disease (CVD) after index in **male** children <18 years with Leukemia, CNS malignancies, Lymphoma, and Testis cancer that received treatment according to standard protocols.

	CNS tumors n = 701	Leukemia n = 697	Lymphoma n = 533	Testis cancer n = 362
Age at index median (IQR)	14 (6-19)	9 (3-16)	18 (13-22)	22 (19-23)
Time from index to CVD years median (IQR)	14.8 (1.5-31.4)	1.0 (0.2-7.6)	11.7 (0.5-27.7)	14.1 (1.0-27.6)
Median age at CVD years median (IQR)	27.7 (16.1-45.0)	14.3 (7.0-23.1)	28.4 (19.0-45.1)	35.3 (23.3-47.9)
**Cancer treatmentc** n (%)	679 (96.9)	617 (88.5)	424 (79.5)	362 (100.0)
Radiotherapy	612 (90.1)	159 (25.8)	225 (53.1)	243 (67.1)
Anthracycline	0 (0.0)	599 (97.1)	381 (89.9)	0 (0)
Other drugs	568 (83.7)	617 (100.0)	215 (50.7)	276 (76.2)
**Cardiovascular diseases** n (%) **Time to CVD and Age at CVD** years median (IQR)				
Hypertension (HTN)	180 (25.7)	184 (26.4)	145 (27.2)	137 (37.8)
Time to HTN	33.4 (20.8-43.0)	1.8 (0.2-16.7)	31.7 (16.5-39.4)	29.2 (19.1-38.3)
Age at HTN	47.6 (35.0-56.6)	12.1 (4.1-27.3)	47.7 (35.8-55.6)	48.0 (40.0-57.3)
Arrhythmias (ARY)	127 (18.1)	98 (14.1)	126 (23.6)	82 (22.7)
Time to ARY	20.6 (2.0-37.0)	3.0 (0.5-18.1)	30.8 (18.2-38.1)	23.1 (10.9-36.2)
Age at ARY	30.4 (13.9-51.8)	18.3 (11.6-27.3)	48.7 (36.5-57.6)	44.4 (32.7-55.4)
Ischemic heart diseases (IHD)	60 (8.6)	27 (3.9)	120 (22.5)	44 (12.2)
Time to IHD	38.9 (25.9-47.0)	22.1 (0.4-34.5)	28.6 (21.6-34.9)	28.7 (20.2-38.3)
Age at IHD	53.3 (45.4-64.6)	35.8 (6.4-46.1)	47.3 (41.2-54.4)	50.5 (44.2-61.1)
Heart failure (HF)	61 (8.7)	105 (15.1)	117 (22.0)	30 (8.3)
Time to HF	27.4 (4.7-42.7)	2.3 (0.5-15.2)	25.9 (7.0-36.9)	31.0 (15.3-37.7)
Age at HF	38.7 (21.7-57.9)	16.5 (8.8-25.6)	43.8 (24.0-55.1)	51.1 (39.3-57.0)
Cerebrovascular diseases	296 (42.2)	174 (25.0)	63 (11.8)	39 (10.8)
Time to Cerebrovascular diseases	15.3 (0.6-33.2)	1.0 (0.1-3.8)	25.5 (8.5-38.1)	28.0 (16.6-37.7)
Age at Cerebrovascular diseases	28.5 (15.6-44.9)	15.3 (7.6-23.0)	45.1 (26.7-56.8)	48.2 (35.7-58.9)
Intracranial hemorrhage (ICH)	163 (23.3)	121 (17.4)	21 (3.9)	10 (2.8)
Time to ICH	7.8 (0.2-28.4)	0.8 (0.1-2.2)	16.1 (0.9-33.2)	12.1 (0.8-24.3)
Age at ICH	23.7 (15.0-43.4)	14.2 (7.7-20.3)	33.9 (17.5-45.1)	33.1 (23.3-41.3)
Cerebral infarction (CVA)	102 (14.6)	24 (3.4)	30 (5.6)	15 (4.1)
Time to CVA	30.1 (13.5-38.5)	24.0 (1.7-36.5)	26.2 (22.4-39.3)	35.3 (25.2-46.8)
Age at CVA	43.3 (28.5-53.4)	39.3 (12.2-49.2)	46.7 (38.4-58.5)	53.5 (48.2-63.8)

Table 6. All cause and cardiovascular mortality outcomes after the first cardiovascular disease in children <18 years with Leukemia, CNS malignancies, Lymphoma, and Testis cancer that received treatment according to standard protocols.

	**CNS tumors n = 1242**	**Leukemia n = 1189**	**Lymphoma n = 839**	**Testis cancer n = 362**
Follow-up time after CVD years, median (IQR)	5.2 (0.5-13.4)	2.9 (0.0-9.9)	6.2 (1.8-13.1)	8.5 (2.9-14.8)
**All-cause mortality** n (%)				
All-cause mortality	526 (42.4)	477 (40.1)	239 (28.5)	56 (15.5)
5-year mortality after CVD[a]	407 (39.4)	451 (48.4)	165 (25.7)	32 (14.0)
**Cardiovascular mortality** n (%)				
Cardiovascular mortality	216 (17.4)	243 (20.4)	108 (12.9)	23 (6.4)
5-year CV mortality after CVD[b]	181 (22.4)	236 (33.0)	79 (14.2)	17 (6.8)
**5-year survivors after index cancer**	**CNS tumors n = 974**	**Leukemia n = 666**	**Lymphoma n = 696**	**Testis cancer n = 327**
Follow-up time after CVD years, median (IQR)	8.0 (2.6-15.4)	8.5 (4.3-14.9)	8.3 (3.3-14.6)	9.3 (4.2-16.1)
**All-cause mortality** n (%)				
All-cause mortality	294 (30.2)	83 (12.5)	162 (23.3)	38 (11.6)
5-year CV mortality after CVD[c]	175 (21.8)	57 (10.6)	88 (15.6)	20 (7.9)
**Cardiovascular mortality** n (%)				
Cardiovascular mortality	118 (12.1)	29 (4.4)	77 (11.1)	15 (4.6)
5-year CV mortality after CVD[d]	83 (11.7)	22 (4.4)	48 (9.2)	9 (3.7)

Abbreviations: CAYAs=children, adolescents, and young adults. CNS=Central nervous system. CV=cardiovascular. CVD=cardiovascular diseases. IQR=interquartile range. Na=not applicable. n=numbers.
[a] 208 CNS tumors, 258 leukemia, 198 lymphoma and 91 testis cancer censored due to follow-up shorter than 5 years.
[b] 434 CNS tumors, 473 leukemia, 284 lymphoma and 112 testis cancer censored due to follow-up shorter than 5 years, or mortality from other causes.
[c] 172 CNS tumors, 129 leukemia, 132 lymphoma and 74 testis cancer censored due to follow-up shorter than 5 years.
[g] 264 CNS tumors, 164 leukemia, 172 lymphoma and 85 testis cancer censored due to follow-up shorter than 5 years, or mortality from other cause.

Table 7. Sociodemographic factors for females and males CAYAs and Controls.

	CAYAs		Controls		
	Female	Male	Female	Male	**p-value**[a]
n (%)	8947 (71.8)	3508 (28.2)	35 449 (76.2)	11 077 (23.8)	
Age at index years median (IQR)	22 (19-23)	16 (8-21)	22 (20-23)	18 (11-22)	<0.0001
**Living conditions at index**					
**Part of Sweden** n (%)					<0.0001
North	946 (10.6)	442 (12.6)	3987 (11.2)	1500 (13.5)	
Central	2924 (32.8)	1367 (39.1)	11 096 (31.3)	4076 (36.8)	
South	5058 (56.6)	1686 (48.2)	20 366 (57.4)	5501 (49.7)	
**Inhabitant/km^2 in municipal** n(%)					0.58
<15	748 (8.4)	420 (12.0)	3095 (8.7)	1405 (12.7)	
15 to 2241	6957 (77.9)	2639 (75.5)	27549 (77.7)	8363 (75.5)	
≥ 2241	1223 (13.7)	436 (12.5)	4805 (13.6)	1309 (11.8)	
**Median income**[e] (SEK) n (%)					<0.0001
>363 000	1185 (13.3)	575 (16.4)	4394 (12.4)	1619 (14.6)	
285 000 to 363 000	6912 (77.4)	2535 (72.5)	27 681 (78.1)	8080 (72.9)	
<285 000	831 (9.3)	385 (11.0)	3374 (9.5)	1378 (12.4)	
**Gini coefficient**[e] n (%)					0.12
> 0.42	840 (9.4)	389 (11.1)	3194 (9.0)	1127 (10.2)	
0.31 to 0.42	7287 (81.6)	2697 (77.2)	29 026 (81.9)	8612 (77.8)	
<0.31	801 (9.0)	409 (11.7)	3229 (9.1)	1338 (12.1)	
**Proximity to hospital** n (%)					0.15
<30 km (19 miles)	7510 (84.12)	2873 (82.20)	29 701 (83.8)	8874 (80.1)	
30 to 100 km	1381 (15.47)	600 (17.17)	5593 (15.8)	2100 (19.0)	
>100 km (62 miles)	37 (0.41)	22 (0.63)	155 (0.4)	103 (0.9)	
**Medical leave** n (%)	6591 (73.7)	1666 (47.5)	28 022 (79.1)	7195 (65.0)	<0.0001
Sick leave >180 days n (%)	4003 (44.7)	921 (26.3)	15 683 (44.2)	3241 (29.3)	0.02
Total sick leave + disability pension days median (IQR)	311.0 (78.9-891.4)	230.3 (68.0-637.6)	244.0 (62.0-785.0)	141.0 (39.8-493.0)	<0.0001
**Follow up years** n (%)					<0.0001
0-5	817 (9.1)	892 (25.4)	706 (2.0)	152 (1.4)	
6-10	467 (5.2)	320 (9.1)	1178 (3.3)	300 (2.7)	
11-20	826 (9.2)	479 (13.7)	2632 (7.4)	864 (7.8)	
21-30	1083 (12.1)	529 (15.1)	4250 (12.0)	1627 (14.7)	
31-40	1915 (21.4)	538 (15.3)	8415 (23.7)	2364 (21.3)	
41-50	2625 (29.3)	443 (12.6)	12 030 (33.9)	3394 (30.6)	
51-60	1118 (12.5)	249 (7.1)	5734 (16.2)	2104 (19.0)	
>61	96 (1.1)	58 (1.7)	504 (1.4)	272 (2.5)	
Years of follow up median (IQR)	38.0 (22.7-47.1)	22.1 (5.7-38.6)	41.5 (31.2-48.9)	41.8 (30.1-50.0)	<0.0001
**Age at study end years** n (%)					<0.0001
0-5	152 (1.7)	187 (5.3)	6 (0.0)	11 (0.1)	
6-10	138 (1.5)	180 (5.1)	22 (0.1)	27 (0.2)	

11-20	382 (4.3)	480 (13.7)	147 (0.4)	170 (1.5)	
21-30	709 (7.9)	643 (18.3)	1442 (4.1)	686 (6.2)	
31-40	835 (9.3)	459 (13.1)	2977 (8.4)	1175 (10.6)	
41-50	998 (11.2)	507 (14.5)	4200 (11.8)	1846 (16.7)	
51-60	1702 (19.0)	480 (13.7)	7854 (22.2)	2809 (25.4)	
61-70	2459 (27.5)	365 (10.4)	11 190 (31.6)	2718 (24.5)	
≥71	1572 (17.6)	207 (5.9)	7611 (21.5)	1635 (14.8)	
Median age at study end (IQR)	58.7 (41.3-68.1)	36.4 (21.5-54.3)	62.1 (51.1-69.8)	57.1 (45.7-66.7)	<0.0001

12,455 child, adolescent, and young adult patients with cancer and cardiovascular disease and 46,526 controls encompassing all cancer patients in Sweden under the age of 25 years from January 1958 to December 2021.Patients included were between <1 years and 24 years at the time of their index cancer diagnosis with 1:5 matched controls based on age sex and place of residence.
Abbreviations: CAYAs=children adolescents and young adults. IQR=interquartile range. n=numbers. SEK=Swedish crown
CAYAs: Data on living conditions is missing for 32 individuals, and data on place of birth is missing for 2 individuals.
[a] p-values apply to comparisons between all CAYAs and controls for outcomes, regardless of sex.
